# Divergently Transcribed ncRNAs in *Escherichia coli*: Refinement of the Transcription Starts Assumes Functional Diversification

**DOI:** 10.3389/fmolb.2021.610453

**Published:** 2021-03-03

**Authors:** Sergey Kiselev, Natalia Markelova, Irina Masulis

**Affiliations:** Department of Functional Genomics and Cellular Stress, Institute of Cell Biophysics Russian Academy of Sciences, Pushchino, Russia

**Keywords:** divergent transcription, non-coding RNAs, genome annotation, REP elements, secreted RNAs

## Abstract

Non-coding regulatory RNAs (ncRNAs) comprise specialized group of essential genetically encoded biological molecules involved in the wide variety of cellular metabolic processes. The progressive increase in the number of newly identified ncRNAs and the defining of their genome location indicate their predominant nesting in intergenic regions and expression under the control of their own regulatory elements. At the same time, the regulation of ncRNA’s transcription cannot be considered in isolation from the processes occurring in the immediate genetic environment. A number of experimental data indicate the notable impact of positional regulation of gene expression mediated by dynamic temporal DNA rearrangements accompanying transcription events in the vicinity of neighboring genes. This issue can be perceived as particularly significant for divergently transcribed ncRNAs being actually subjected to double regulatory pressure. Based on available results of RNAseq experiments for *Escherichia coli*, we screened out divergent ncRNAs and the adjacent genes for the exact positions of transcription start sites (TSSs) and relative efficiency of RNA production. This analysis revealed extension or shortening of some previously annotated ncRNAs resulting in modified secondary structure, confirmed stable expression of four ncRNAs annotated earlier as putative, and approved the possibility of expression of divergently transcribed ncRNAs containing repetitive extragenic palindromic (REP) elements. The biogenesis of secreted ncRNAs from divergently transcribed *ffs*, *chi*X, *ral*A, and *ryh*B is discussed taking into account positions of TSSs. Refinement of TSSs for the neighboring genes renders some ncRNAs as true antisense overlapping with 5′UTR of divergently transcribed mRNAs.

## Introduction

**FIGURE 1:**
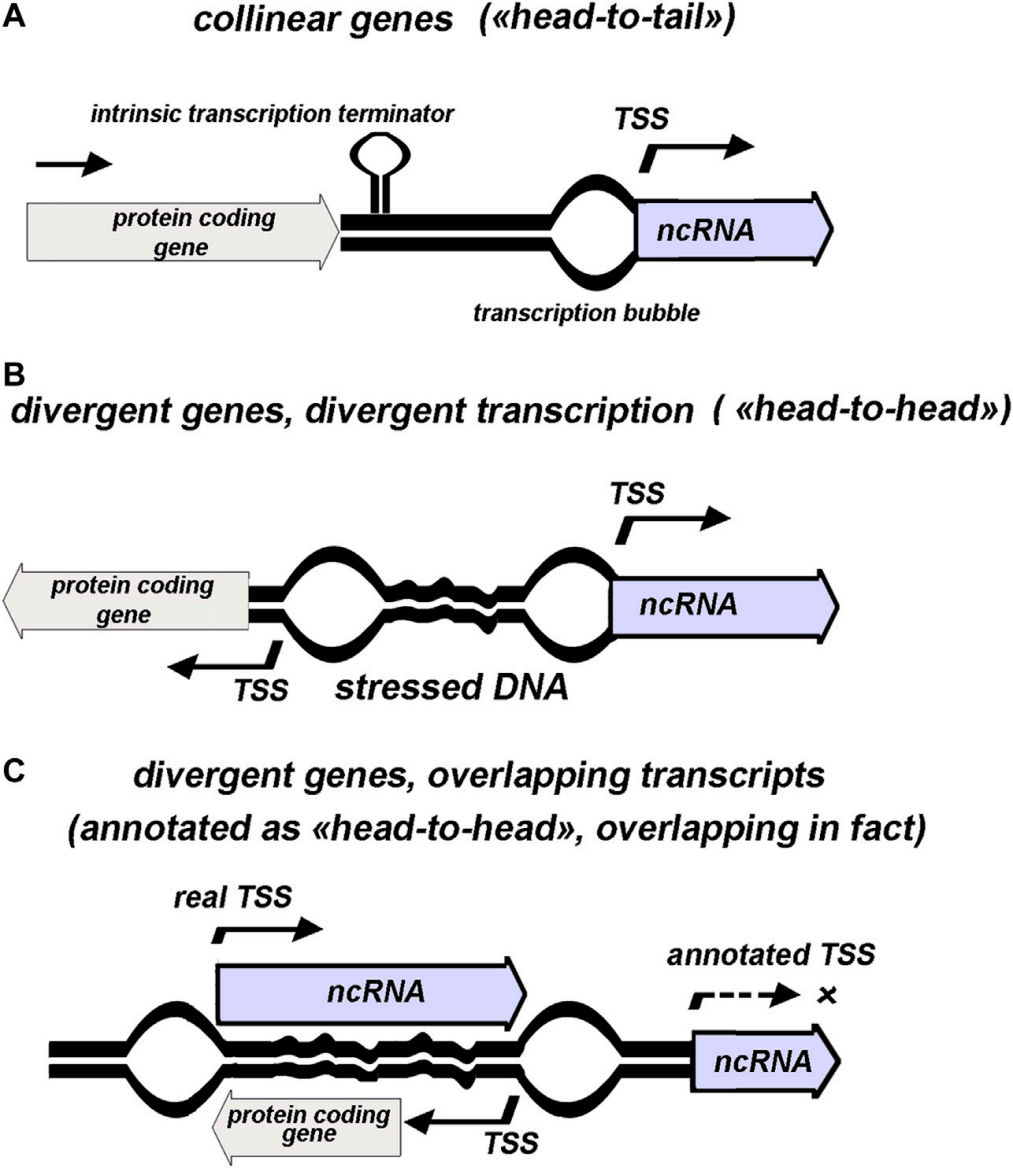
Schematic presentation of various genomic contexts for ncRNAs annotated as separate transcription units. 5′ end of ncRNA assumed to coincide with transcription start site (TSS). “Stressed DNA” and positions of “transcription bubbles” illustrate topology-mediated effects.

The generally accepted classification of non-coding regulatory RNAs (ncRNAs) is primarily based on their genomic positioning relatively to the target gene subjected to regulation. Thus *cis*- and *trans*-acting subtypes are described (reviewed in Waters and Storz, 2009), implying that the first control the expression of closely located gene (primarily via antisense mechanism and base pairing protruding over 43 to 650 bp) (Brantl, 2007) while *trans*-encoded sRNAs are confirmed to have from one to dozens remote targets detected by means of biochemical and bioinformatics approaches (Vogel and Wagner, 2007; Hör et al., 2020). Assignment of ncRNAs as powerful and numerous class of metabolic regulators stimulated a flow of works aimed to discover and comprehensively characterize physiologically relevant products. For a long time, the dominated strategies exploited 1) the search of conservative intergenic regions (IGRs), 2) the presence of transcription initiation and termination signals, and 3) the ability of potential ncRNA to adopt stable secondary structure (Altuvia, 2007; Backofen and Hess, 2010). Predicted candidates were experimentally verified using hybridization and reverse transcription approaches. This allowed revealing major ncRNAs as master regulators with defined biological function (Argaman et al., 2001; Dutta and Srivastava, 2018). Selective cloning strategy aimed to hound down small ncRNAs (sRNAs) (Kawano et al., 2005) and high throughput sequence facilities significantly enriching ncRNAs community (Shinhara et al., 2011; Barquist and Vogel, 2015) including independently encoded genes and ncRNAs, originated by cutting-out from mRNAs. In *E. coli*, the observation of non-uniformity in decay rate of entire mRNAs and their internal fragments led to the finding of 58 novel putative ncRNAs partially or completely embedded into the coding regions (Dar and Sorek, 2018). Although recently described ways to produce ncRNAs from 5′ and 3′ untranslated regions of coding sequences contribute to the whole spectrum of riboregulators (Carrier et al., 2018), their transcription from intergenic regions remains one of the primary sources (Adams and Storz, 2020). The mode of ncRNA’s biogenesis—from IGRs by means of transcription from specified promoter or from coding genomic region and adjacent loci mediated by RNAse E—implies the involvement of different molecular mechanisms in the regulation of their abundance. ncRNA excised from mRNA precursor follows the pathways implicated in the control of parental RNA synthesis, although additional level of regulation may be imposed by accessibility of distinct structural motifs to RNAses. IGR-derived ncRNA possesses individual control elements including promoter, terminator, and transcription factor binding sites. ncRNAs encoded within IGRs undergo more strict purifying selection if compared to non-coding regions containing promoters or terminators only (Thorpe et al., 2017). Genome**-**wide analyses encompassing near 900 sRNAs from 13 bacterial species revealed meaningful enrichment of long conserved IGRs by non-coding sRNAs (Tsai et al., 2015). Since these sRNAs may be considered as “self-sufficient” genetic elements, one might expect their conservation between different species and evolutionary distant taxonomic bacterial groups. Nevertheless, comparative analysis highlighted substantial deviation in phylogenetic distances calculated for sRNAs and for protein-coding sequences (Lindgreen et al., 2014). Thus, ncRNAs undergo their own way of emergency and evolution still remaining one of the most obscure matters. It was proposed that promoters for ncRNAs could arise from transcriptional noise in terms of new promoter spontaneously erupting in IGR and conferring beneficial properties (Jose et al., 2019). Such RNAs must go “trial and error” path for the adaptation to the regulatory networks of the cell being probed for the compatibility with different targets among mRNAs and RNA-binding proteins. ncRNA’s implementation in metabolic circuits may occur as coevolution with RNA-binding proteins (ChiX, CyaR, FnrS, MicA, SgrS, DsrA, and MicC) or like prevenient gain of novel ncRNAs searching for a target (ArcZ, MicF, OmrA, OxyS, and RprA) (Peer and Margalit, 2014). The lack of the initial destination for the interaction with a specific partner may partially explain the phenomenon of “multi-target” action of many *trans*-encoded ncRNAs. Other possible mechanisms giving rise to the novel ncRNAs may be horizontal gene transfer and duplication of preexisting ncRNAs followed by the acquisition of new functions (Dutcher and Raghavan 2018). Preferential location of ncRNAs in IGRs assumes that, apart from involvement of their own regulatory elements, transcription of neighboring genes may contribute to the maintenance of their expression at appropriate level. Using *lac* promoter-based reporter system (Bryant et al., 2014) or barcoded construct (Scholz et al., 2019), it was demonstrated that transcriptional output varies in the range of 300- or 20-fold, respectively, depending on the local genetic environment. This positional regulation via mechanistic properties of given DNA segment also may affect the “decision” for certain RNA to be expressed from the certain chromosomal region. The influence of genomic context is expected to be quite different for RNAs having collinear or divergent orientation, respectively, to the adjacent gene accounting concomitant conformational effects induced by transcription (Figure 1). The model of twine-supercoiling domains postulates the formation of partially unwound DNA regions behind transcription complexes and accumulation of positive supercoils on the front line (Wu et al., 1988). Since initiation of transcription was shown to be sensitive to DNA superhelical state (Lim et al., 2003; Peter et al., 2004; Zhi et al., 2017; Dorman, 2019), the expression of closely located transcription units in fact may be considered as context-encoded regulatory level implicated in the control of ncRNAs synthesis. Gene expression profiling revealed altered response of divergent genes to the global changes in superhelical density induced by DNA gyrase inhibitor norfloxacin indicating the combined effect of local and global DNA conformational changes on transcription (Masulis et al., 2015). In this respect for the case of divergently transcribed ncRNAs, the relative positions of promoter regions and the rate of their utilization by RNAP appear to be critical for the final transcriptional output. As for any pair of divergent transcriptional units, transcript level for each counterpart is expected to be a result of mutual effects mediated by competition for enzyme binding or by means of non-contact structural DNA deformation or by both mechanisms concurrently. Here, we addressed if codirected or divergent positioning of known ncRNAs has any preference and if there is any bias—antagonistic, synergistic, or neutral—between the expression of ncRNAs and their divergent partners.

Totally, 125 genes with established or proposed assignment to non-coding small RNAs are annotated in RegulonDB to date (Santos-Zavaleta et al., 2019). 12 of them overlap with 5′ or 3′ terminal parts of protein-coding sequences assuming that transcription of these RNAs *a priori* is superimposed with read-through of parental mRNA. Based on annotation, 61 codirected and 64 divergently transcribed ncRNAs are revealed in *E. coli* genome demonstrating the lack of predominant orientation of ncRNAs relatively to the adjacent genes—“head-to-tail”, “head-to-head”, or overlapping over 5′ termini (Figure 1). Unambiguous attribution of ncRNA as an independent transcriptional unit and its assignment to one of the three types of relative arrangement with respect to the neighboring gene are fraught with some deceptions arising from incompleteness of our knowledge regarding the exact positions of promoters and terminators (Figure 1C). Thus, meta-analysis of data obtained using high-throughput RNAseq and tiling arrays allowed defining true *bona fide* small RNA as a product of gene not overlapping any other genes from the opposite strand (Liu et al., 2018). According to these requirements, antisense RNAs, UTR-processed products, and transcripts derived from premature termination (riboswitch) were sifted out from almost 500 previously proposed staphylococcal ncRNAs resulting in only 46 *bona fide* representatives with high degree of conservation within *Staphylococcaceae* family. Thus, the displacement of TSS into the coding region of the neighboring gene compels to reconsider categorization of ncRNA as *cis*-acting or *trans*-acting. Some cases of this type revealed for *E. coli* ncRNAs are discussed below.

**FIGURE 2:**
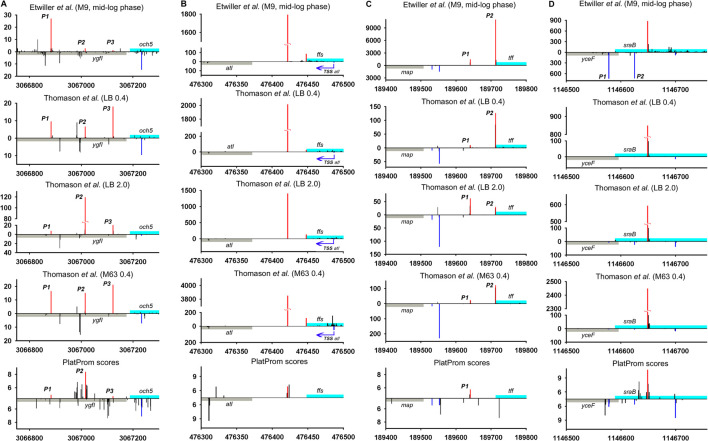
Mapping of TSSs for divergently transcribed ncRNAs according to RNAseq data (Thomason et al., 2015; Ettwiller et al., 2016) and computational prediction (Shavkunov et al., 2009). X axis: coordinates in *E. coli* genome; Y axis: average number of normalized counts with perfect matching for given growth condition. For PlatProm predictions absolute score values are indicated on Y axis. RNA denoted according to the annotation in RegulonDB is shown in cyan; adjacent divergent gene is indicated in gray. Bars above and below X axis correspond to the upper and low DNA strands, respectively. Exactly matching TSSs for ncRNAs are presented in red and for divergently transcribed gene are indicated in blue. Transcriptional landscapes upstream and downstream relatively to the genomic regions depicted in Figure 2 are shown in Supplementary Figure S2.

We juxtaposed the location of TSSs predicted by promoter-search algorithm PlatProm (Shavkunov et al., 2009) and experimentally found 5′ termini of ncRNAs and divergently transcribed neighboring genes of *E. coli* using data sets available from Thomason et al. (2015) and Ettwiller et al. (2016). PlatProm was developed to search for potential TSSs accounting sequence features of experimentally characterized *E. coli* promoters. It evaluates each position in the genome for the ability to serve as transcription start assuming essential promoter textual elements formalized in weight matrixes. The score value reflecting similarity with “ideal” promoter is considered as the quantitative measure of transcriptional capacity for given position in the genome (Shavkunov et al., 2009). The data of Thomason et al. (2015) also allow estimating the expression level of ncRNAs under various conditions. The positions of primary TSSs in superimposed transcriptional profiles are in general in good agreement with each other (Figure 2) confirming the highest value and objectivity of such data for the reconstruction of the genome-wide transcriptional profile as an integral fingerprint of bacterial physiological status in given growth conditions.

### Evidence and Objections

#### Upshift or Downshift of sRNA 5′ End

RNAseq data allowed verifying the exact positions of transcription starts for divergently transcribed ncRNAs (Supplementary Table S1). Although the coordinates of primary TSSs for the majority of ncRNAs are in good agreement with their 5′ ends mapped in RegulonDB, the overall transcriptional landscape of near 30 genomic loci needs some corrections. The most notable examples are considered here. Thus, for G0-8898 putative ncRNA annotated as “phantom gene” TSS seems to be located 27 bp upstream from 5′ end indicated in RegulonDB, (Supplementary Table S1) for *och*5 this shift is 65 bp, and for *ffs* it is 26 bp (Figure 2A,B; Supplementary Figures S2A,B). The opposite deviation toward 3′ end was observed for *rse*X, *ytf*L5′ (Supplementary Table S1), *sra*B (Figure 2D; Supplementary Figure S2D) genes—TSSs were moved downstream by 29, 43, 60, and 65 bp, respectively, resulting in shortening of corresponding ncRNAs. Additional TSS exactly matching computational predictions by PlatProm and prevailing in stationary phase was found for *tff* (Figure 2C; Supplementary Figure S2C). Thus, *map*-*tff* genomic loci may produce two divergently transcribed growth-phase dependent ncRNAs differing in length by 71 nt. Nor synergistic or cooperative effect in the expression of *map* and *tff* was observed assuming their autonomous regulation. For *crp*T, double-side deviation was revealed—weak promoter with start point was pushed 164 bp upstream to the annotated start and primary TSS shifted 6 bp downstream (Supplementary Figure S1B). Second-order 5′-extended CrpT RNA with considerable expression level only at stationary phase may serve as antisense for yheU mRNA forming the duplex with 5′UTR (promoter P2). RydC, one of the most abundant and early described sRNAs implicated in the wide spectrum of regulatory networks (Antal et al., 2005) possesses upstream TSS remote from the primary start by 96 bp which enlarges the secondary putative sRNA by a factor 3.

**FIGURE 3:**
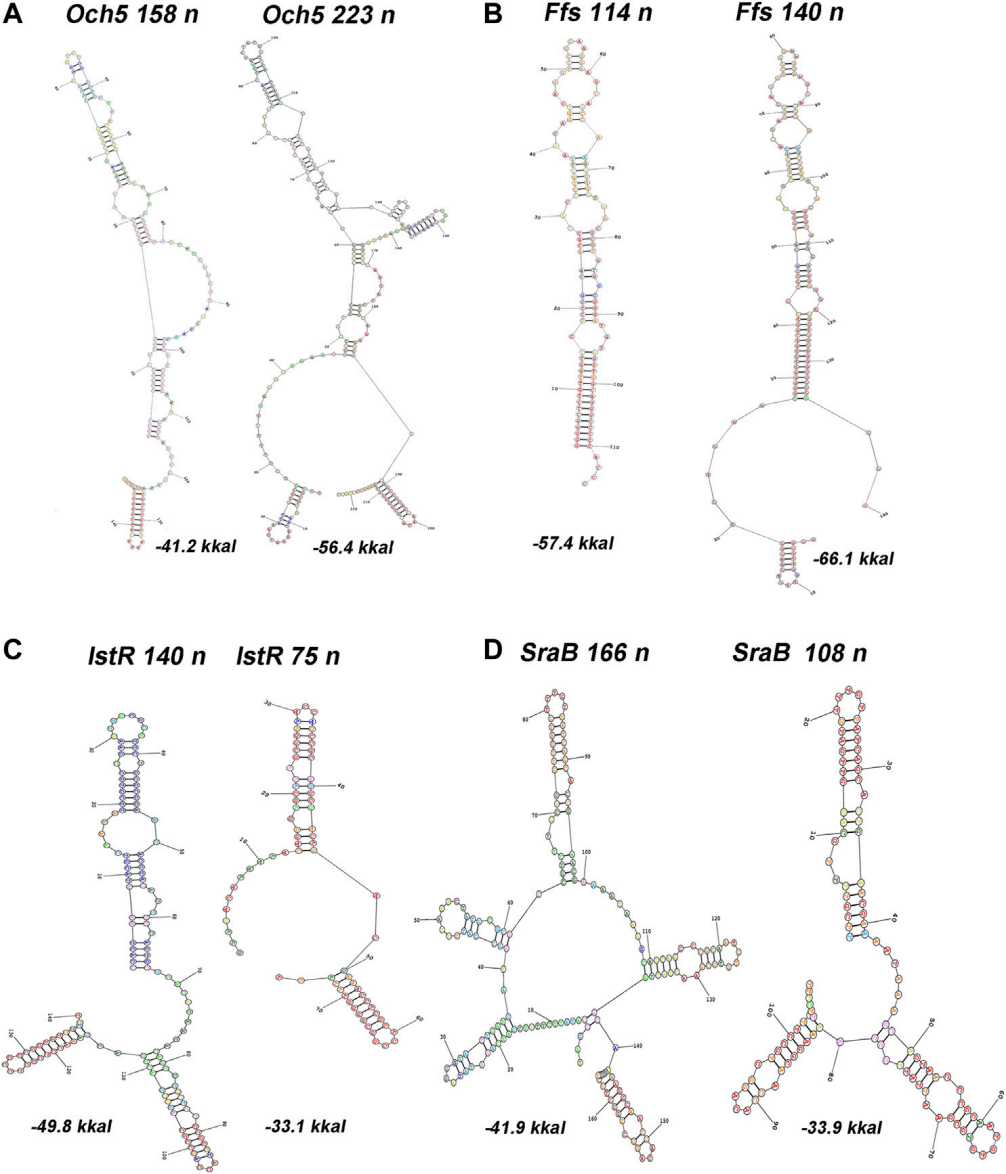
Predictions of secondary structures for 5′-extended or shortened ncRNAs. RNA structure was modeled using server http://rna.urmc.rochester.edu/RNAstructureWeb/Servers (Reuter and Mathews, 2010). Transcripts originating from two TSSs, one corresponding to annotated start (RegulonDB) and the other inferred from RNAseq data (Thomason et al., 2015), were subjected as a query using default parameters. Annotated position of 3′-end was assumed for both variants. Energy of folding is indicated below the structures.

The upshift as well as downshift of ncRNA’s 5′ ends if compared to previously accepted positions may account for changing of folding properties and thus selectivity to RNA binding proteins and mRNA targets. Extension or shortening of ncRNA may result in 1) considerable rearrangements of folding pattern (Och5, Figure 3A), 2) addition of a new structural motif like when it happens in the case of Ffs and IstR while the “core” ncRNA’s structure remains the same (Figures 3B,C), or 3) elimination of intramolecular stem-loop structures if TSS appears to be pushed downstream (SraB, Figure 3D). Newly attached or removed structural details in ncRNA’s architecture may serve as anchors for the interaction with partner molecules, thus determining the spectrum of ncRNA’s targets. Noteworthy enlargement or trimming of ncRNAs deduced from refined TSS position does not noticeably change energy of folding and specific impact of nucleotides in RNA stability. SraB and IstR are the exceptions showing increased nucleotide impact in free energy of folding by 26% and 23.8% correspondently when shortened from 5′ end, respectively, to the annotated length. SraB belongs to the classic type of divergently transcribed overlapping ncRNAs, firstly discovered by Argaman et al., 2001, confirmed by Northern blot and *in vitro* transcription assay, and conserved in *Salmonella typhimurium*, *Klebsiella pneumoniae*, and *Yersinia pestis*. Although in early experiments the expression of *sra*B was proposed to be growth phase dependent and was detected in late stationary phase in LB but not in the mineral medium, RNAseq data pointed out stable production of this RNA in 5′-truncated form in exponential phase in LB as well as in M63 with threefold increase in minimal medium. Surprisingly, in virulent *S. typhimurium* strain, SL1344 *sra*B TSS corresponds to TSS revealed for *E. coli* in Thomason et al. (2015) and Ettwiller et al. (2016), and the length of homologous RNA is expected to be 99 nucleotides, close to that of “shortened” *E. coli* derivative (data from Supplementary Table S8 in Ramachandran et al., 2014). *S. typhimurium* SraB is shown to be significantly suppressed by ppGpp (Ramachandran et al., 2014) and is proposed to confer resistance to egg albumin (Jiang et al., 2010). The discrepancy in the positioning of SraB 5′-end was probably caused by the presence of deceptively “attractive” canonical sigma70 promoter related to the previously annotated TSS. Downshifted transcription start highlighted by RNAseq on the contrary corresponds to poorly defined promoter although it was indicated as “predicted” in RegulonDB (Santos-Zavaleta et al., 2019). It remains questionable why of the two promoters only one possessing less similarity to consensus is engaged in RNA synthesis. One of the possible explanations may assume collision effects due to transcription initiated at *yce*F P1 promoter (Figure 2D, data set of Ettwiller et al. (2016)). At the same time, *sra*B transcription is insensitive to the activity of divergent overlapping promoter *yce*F P2. Another data set used in this study (Thomason et al., 2015) has not confirmed transcriptional activity of *yce*F while the position of SraB TSS perfectly coincides for all RNAseq experiments and computational scanning by PlatProm (Figure 2D; Supplementary Figure S2D).

Ffs ncRNA overlaps with divergent *atl* transcript by 65 bp although the level of *atl* transcript appeared to be negligible (Figure 2B; Supplementary Figure S2B). Ffs named also 4.5S is one of the most “deserved” among ncRNAs in the historical aspect due to its function as an essential component of signal recognition particles (SRP). Motif 5′ACC​AGG​TCA​GGT​CCG​GAA​GGA​AGC​AGC responsible for the formation of apical helix structure was shown to be critical for the interaction with Ffh protein and conserved in bacteria and even high organisms (Peterson and Philips, 2008). Extension by 26 bases from 5′ end does not interfere with the overall folding pattern although additional stem-loop structure appears at the terminus (Figure 3B). This appendage as a consequence of TSS upshift is conserved among *Enterobacteriaceae* including *Escherichia*, *Shigella*, *Salmonella*, *Enterobacter*, *Citrobacter*, *Klebsiella*, *Kluyvera*, *Kosakonia*, and *Trabulsiella* as confirmed by Microbial Nucleotide BLAST when extended *ffs* gene was submitted as a query. It is noticeable that in spite of location on plus or minus strand *ffs* retains opposite orientation to the neighboring gene. The relative efficiency of transcription in the pair *ffs*P1 /*atl *P1 for the three experimental conditions used in Thomason et al. (2015) implies antagonistic rather than supporting effect of atlP1 on ffsP1 activity consistent with collision model (Callen et al., 2004).

Duplicated promoters are likely to regulate the expression of *rye*G having two TSSs separated by 26 bp. The amount of corresponding ncRNAs depends on the growth conditions. Both short and long RNAs are consistently activated in stationary phase along with the transcription of the adjacent *tfa*S gene demonstrating cooperative behavior (Supplementary Figure S1A, data set of Thomason et al., 2015). Indeed, the distances between TSSs related to promoters P1 and P2 and the start point of TfaS mRNA (57 and 83 bp correspondently) allow alternate or simultaneous binding of RNA polymerase to transiently unwound DNA and transcription initiation in opposite directions. Two variants of RyeG RNA of 194 and 220 nt in length having identical folding in the core part of the molecule differ in the structure of 3′terminus (Supplementary Figure S3) that may result in altered recognition of their targets.

Thus, the same genomic region may be the source of two or more specious ncRNAs differing in length, shape, and stability, thereby assuming to expand the whole spectrum of related functions.

### Potential Antisense Transcripts Inferred From TSSs Mapping

**FIGURE 4:**
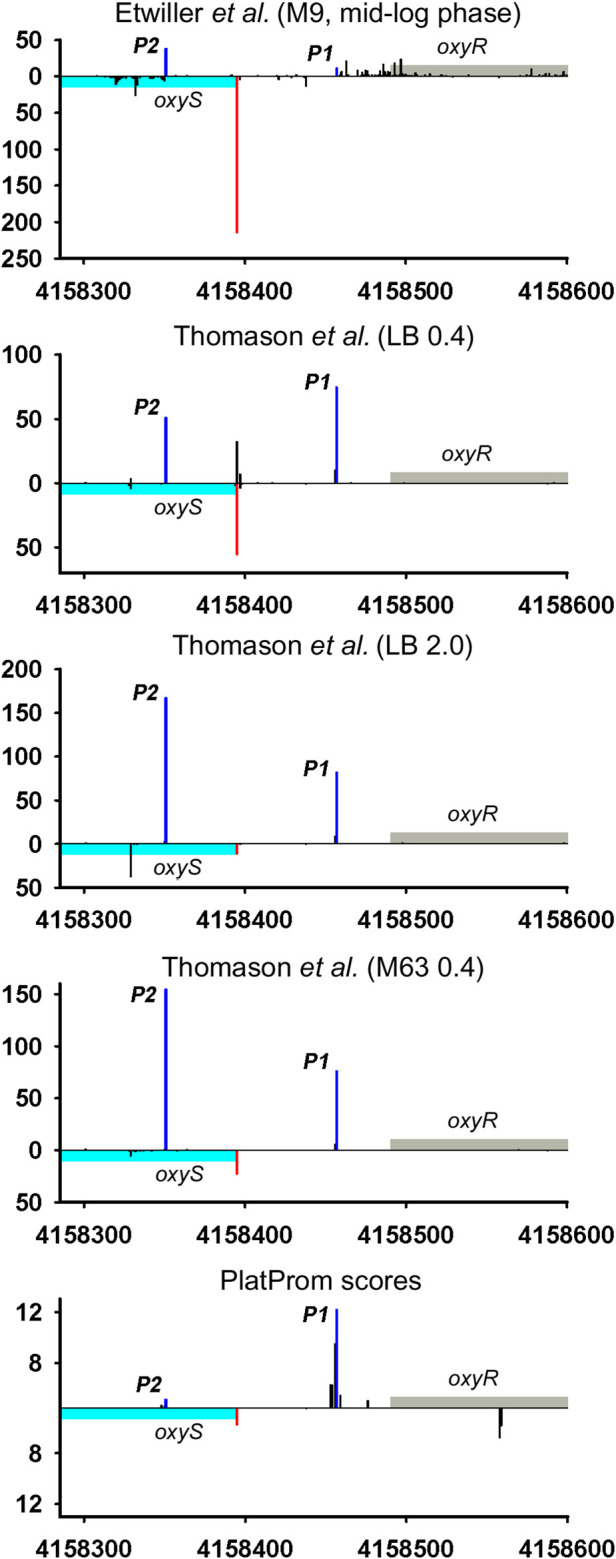
Mapping of TSSs for *oxy*S-*oxy*R genomic region according to RNAseq data (Thomason et al., 2015; Ettwiller et al., 2016). All designations are the same as in Figure 2. Transcriptional landscape upstream and downstream relatively to the genomic region depicted in Figure 4 is shown in Supplementary Figure S4.

Divergently encoded ncRNAs may be added to the family of antisense ncRNAs accounting for newly discovered or corrected positions of TSSs for adjacent divergent genes. Thus, OxyS may overlap 5′UTR of its *vis-*à*-vis oxy*R by 41bp. Described in 1997 (Altuvia et al., 1997), this RNA for more than 20 years has never ceased to amaze researchers with the variety of its functions. Annotated TSS was concurrently detected in all RNAseq experiments and exactly predicted by PlatProm algorithm (Figure 4; Supplementary Figure S4). Assuming the activity of sole P1 *oxy*R promoter OxyS may be defined as typical *bona fide* ncRNA although obvious activity of P2 promoter coming to be major in stationary phase and in mineral medium allows to ascribe to OxyS potential role of antisense RNA.

Similarly, MicC over its entire length may serve as antisense for *omp*N mRNA having long 5′UTR with TSS remote from initiation codon by 408 bp (Supplementary Table S1). In the pair, *mic*A/*lux*S highly expressed *mic*A sRNA is overlapped with 5′UTRs of one of two *lux*S-related mRNAs. MicA is involved in a number of RNA-RNA interactions in *trans* presumably with mRNAs related to cell-envelop stress (PhoP, LamB, OmpA, OmpW, Tsx, OmpX, FimB) (Mihailovic et al., 2018). For example, MicA is capable of forming stable duplex with OmpA mRNA as confirmed *in vitro* using SPR technique (Vincent et al., 2013). Overlapping of ncRNAs with 5′UTRs of divergent mRNAs allowing ascribing to the non-coding transcripts potential antisense functions may be proposed for the pairs G0-10702/YhcC (15 bp duplex), G0-10703/yhcG (128 bp), RdlD/ldrD (136 bp), G0-10705/GlnA (63 bp, significant for stationary phase), SymR/SymE (76 bp, entire length), G0-10703/YhcG, ArrS/GadE (68 bp, entire length) (Supplementary Table S1).

Therefore, two types of corrections based on RNAseq data make it possible to consider ncRNA as antisense, identifying a previously unknown TSS for the divergent gene and/or discovering additional TSSs for ncRNA. Transcriptional regulation via multiple promoters is widely exploited in prokaryotes and it seems to be a general principle of promoters evolving in intergenic loci. Thus, from 28 to 40% of experimentally found promoters for mRNA are accompanied by the “second-order” regulatory sites within 250 bp long region upstream to the ORFs (Huerta and Collado-Vides, 2003). The classical example of paired promoters for the transcription of rRNA separated by 120 bp ensures flexible growth rate-dependent regulation of ribosome synthesis (Gourse et al., 1996). *dad*A encoding d-amino acid dehydrogenase is transcribed from three consecutive promoters differing in their activity and selective response to cAMP-CRP (Zhi et al., 1998). In some cases, tandemly arranged promoters may be recognized by RNA polymerase holoenzyme containing alternative sigma-factors and adopted to respond to changing environmental conditions, thus providing reserve facilities for transcriptional regulation. For example, AppY mRNA may be initiated at two promoters separated by 76 bp and recognized by sigma70 or sigma38 RNAP (Rangarajan and Schnetz, 2018). Sigma70 and sigma38 dependent TSSs of *nha*A are separated by 141 bp demonstrating differential sensitivity to HNS and elevated sodium concentrations (Dover and Padan, 2001). Promoter duplication may obviously be exploited in bacteria for the fine turning of ncRNA’s expression and the extension of accessibility of divergent mRNAs for regulation via antisense mechanism.

### Divergent ncRNAs as Purveyors of Extracellular exRNAs

Besides indubitable universality of ncRNAs in coordination of intracellular regulatory processes, their role as messenger molecules in cell communications is coming to be more and more appreciable. RNA is exported as small fragments (less than 60 nt in length) in a controlled manner exploiting outer membrane vesicles (OMV) or yet unrevealed secretion machinery (Ghosal, 2018). Among the total pool of extracellular RNAs, ncRNA’s derivatives comprise a substantial fraction along with rRNA’s and tRNA’s generated fragments. Divergently transcribed Ffs RNA contributes to this set being preferentially exported in OMV-free manner in *E. coli* (Ghosal et al., 2015). Its function as external signal molecule in bacterial population is likely to be conserved since homologous ncRNA of *Salmonella enterica* serovar Typhimurium LT2 was detected in OMV-enriched samples (Malabirade et al., 2018). Nevertheless, pathways of processing and secretion of Ffs ncRNA differ in two Enterobacteria—in *E. coli* only short fragment of 29 nt was shown to be exported while in *S. Typhimurium* LT2 full-length transcript enclosed in or bound to OMV was revealed. The involvement of Ffs in intercellular communication is confirmed by the observation of considerable increase (up to 13-fold) in its amount in cultivation medium for mixed bacterial population (*E. coli*—*Paenibacillus bisonicum*) if compared to pure *E. coli* culture (Alikina et al., 2018). Another three extracellular exRNAs originated from divergently transcribed precursors (ChiX, RalA, and RyhB) demonstrated opposite reaction on the presence of competing bacterium resulting in 2- to 20-fold decrease in their secretion. It should be noted that 5′ ends of short fragments of ChiX (5′ ACACCGTCGCTTAAAG) and RalA (5′ GAGGACTGAAGT) revealed in cultural fluid (Alikina et al., 2018) exactly correspond to primary TSSs of parental ncRNAs indicating possible endonuclease cutting out or release of aborted transcripts generated upon initiation of RNA synthesis. RyhB-related exRNA (5′CCG​GGT​GCT​GGC​TTT​TTT​T), and vice versa, may be ascribed to 3′ terminus of full-length ncRNA. 5′-end of Ffs-born intracellular fragment (Alikina et al., 2018; Supplementary Table S1) is shifted down by 11 nt relatively to TSS refined according to the data of Thomason et al. (2015). It means that additional stem-loop structure expected to form in 5′extended transcript (Figure 3B) may exert functional role for nibbling out short RNA. Ffs, ChiX, and RyhB belong to the group of the most abundant ncRNAs with enhanced expression in stationary phase (Thomason et al., 2015) raising the possibility of their involvement in the control of cell density in bacterial population. Otherwise, RalA is accumulated at very low level which makes the presence of its fragment among secreted RNAs rather intriguing, indicating selectivity of export.

### Putative Divergent ncRNAs Containing REP Elements

**FIGURE 5:**
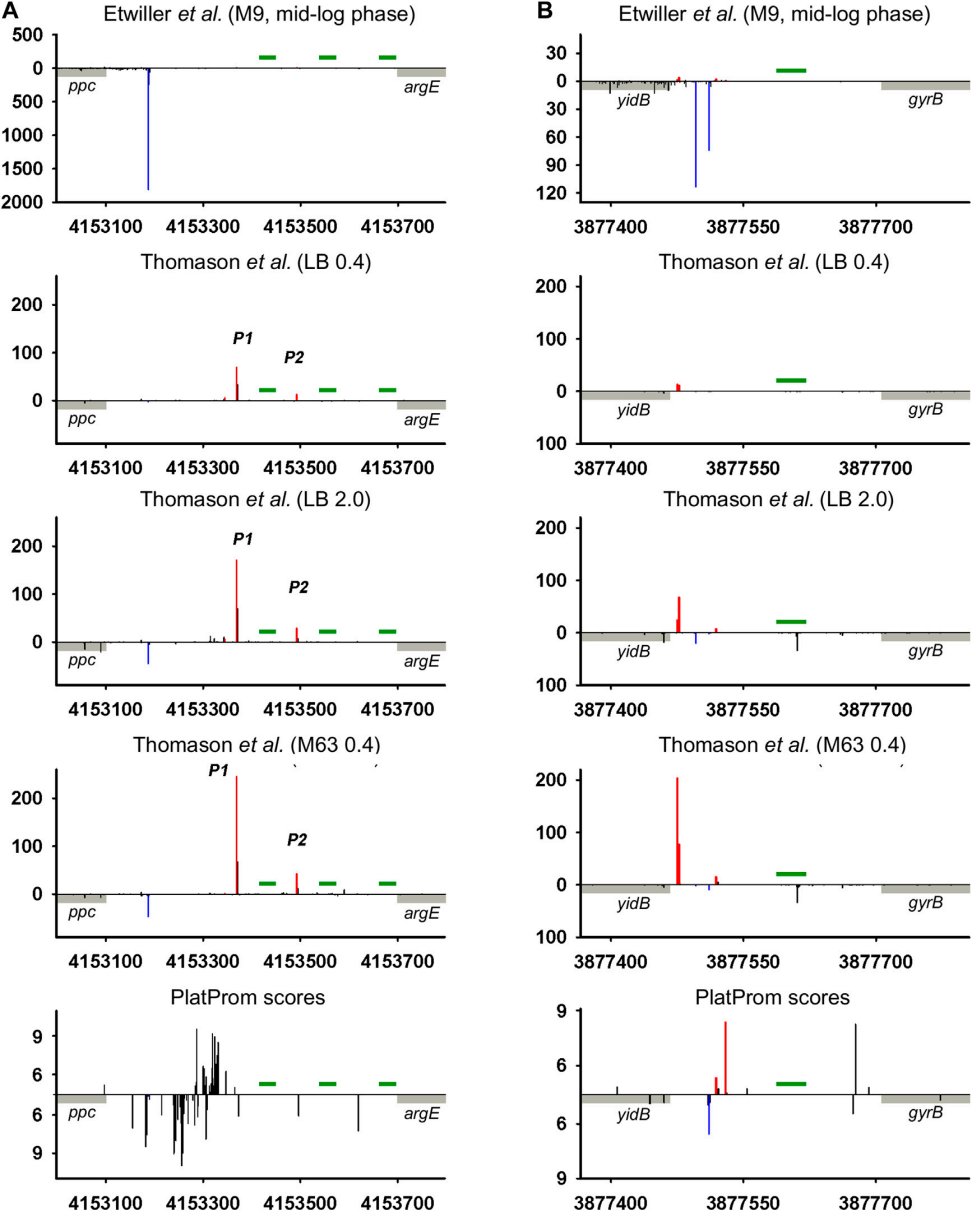
Mapping of TSSs for divergently transcribed REP elements according to RNAseq data (Thomason et al., 2015; Ettwiller et al., 2016) and computational prediction (Shavkunov et al., 2009). X axis: coordinates in *E. coli* genome; Y axis: average number of normalized counts with perfect matching for given growth condition. For PlatProm predictions absolute score values are indicated on Y axis. REP element is denoted in green; adjacent genes are indicated in gray. Bars above and below X axis correspond to the upper and low DNA strands, respectively. Exactly matching TSSs related to divergently transcribed REP sequence are presented in red and for protein-coding genes are shown in blue.

Intergenic regions, along with the fact that they serve as reservoir of ncRNAs, may contain repeating sequences with mirror-symmetry, so called REP (repetitive extragenic palindromic) elements. The transcription of these sequences yielding RNAs with potential regulatory functions is still questionable. REP elements represent conservative universal structural modules of bacterial genomes. Deduced on the base of the alignment of 35 *E. coli* and *S. typhimurium* intergenic regions, REP sequence was proposed to be 35 bp long symmetrical motif GC (g/t)GATGGCG (g/a)GC (g/t)…(g/a)CG (c/t)CTTATC (c/a)GGCCTAC (Stern et al., 1984). Similar stretches homologous to 5′TGC​CGG​ATG​CGG​CGT​AAA​CGC​CTT​ATC​CGG​CCT​AC (Tobes and Ramos, 2005) and 5′GCC​GGA​TGC​GGC​GTG​AAC​GCC​TTA​TCC​GGC​CTA​CGA (Markelova et al., 2016) were found in *E. coli* genome in 290 and 224 copies correspondently without any preferential location between convergent or codirectional genes. Self-complementarity assuming the ability to form hairpin structures in RNA or in negatively supercoiled DNA propelled to discuss the involvement of REP elements in regulation of gene expression (Higgins et al., 1988). In spite of efforts to confirm their role in transcription activation, attenuation, or maintenance of mRNA stability (Higgins et al., 1988), the only function related to premature termination of translation can be considered as experimentally approved (Liang et al., 2015). Thus, REP motif located at a distance no longer than 15 bp from translation termination codon in 3′UTR of mRNA affects the abundance of corresponding protein. At the same time, near 80% of REP elements are pushed down from the 3′ terminus of adjacent gene at a longer distance (Markelova et al., 2016) implying another biological function. It was suggested that REP sequences may be read-through as autonomous transcription units contributing to the family of novel non-coding RNAs (Qian and Adhya, 2017). ncRNA CbsR16 originated from REP-containing repeat-reach region of *Coxiella burnetii* chromosome was described recently (Wachter et al., 2018). Here, we analyzed 79 REP-containing genomic loci separating collinear protein-coding genes for the presence of TSSs providing divergently transcribed RNAs encompassing REP elements. Indeed in 35 cases TSSs were found indicating transcription in opposite direction relatively to the juxtaposed gene (Supplementary Material, List of REP-containing genomic regions). Two representative examples are shown in Figure 5. In the case of *ppc*-*arg*E intergenic region *bona fide* divergent transcription expected to capture 3 and 2 consecutive REP elements was registered (Figure 5A; Thomason et al., 2015). For *yid*B-*gyr*B loci, divergent RNA initiated between two codirected genes seems to be true antisense product complementary to *yid*B 5′UTR (Figure 5B). Weak expression of *bona fide* ncRNA trimmed from 5′ end was detected only for stationary phase and mineral medium. Does the presence of REP elements as a structure-forming motifs impact in the functional properties of corresponding RNAs remains unclear. Obviously, REPs may be included in novel divergently transcribed RNAs if there is no intrinsic terminator between TSS and the 5′-end of REP. Only 16 of 35 REP-containing intergenic regions have T_n_ tracks indicating potential intrinsic terminator between TSS and 5′-end of REP, though not all T-rich blocks are preceded by self-complementary sequence essential for transcription termination. It increases the probability of transcription passing through REP motifs. The distance from TSSs to REP 5′ end varies from 16 to 298 bp allowing also to assume their function in rho-dependent transcription termination proposed in Espeli et al. (2001). The relative positions of TSSs and the borders of REPs admit the possibility of their inclusion in ncRNAs as well as return us to the early hypothesis regarding the role of REPs as transcription punctuation signals. In the last case, REPs may serve to block transcription of divergent ncRNAs, thus determining their length.

### ncRNAs Under the Question—Are They Really Transcribed or Not?

**FIGURE 6:**
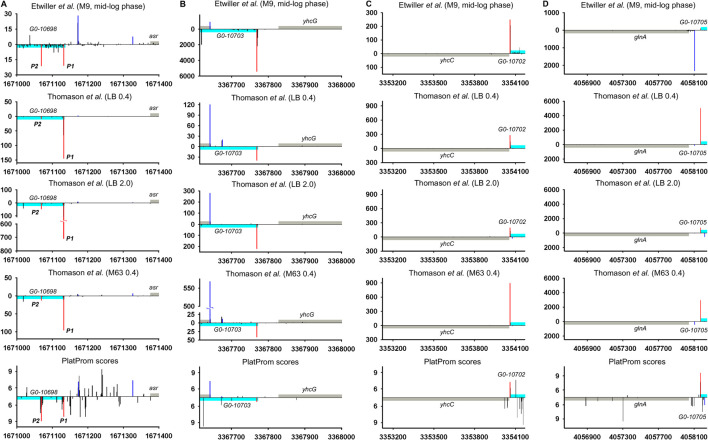
Mapping of TSSs for divergent potential ncRNAs—G0-10698, G0-10703, G0-10702, and G0-10705 according to RNAseq data (Thomason et al., 2015; Ettwiller et al., 2016) and computational prediction (Shavkunov et al., 2009). X axis: coordinates in *E. coli* genome; Y axis: average number of normalized counts with perfect matching for given growth condition. For PlatProm predictions absolute score values are indicated on Y axis. RNA denoted according to the annotation in RegulonDB is shown in cyan; adjacent divergent gene is indicated in gray. Bars above and below X axis correspond to the upper and low DNA strands, respectively. Exactly matching TSSs for ncRNAs are presented in red and for divergently transcribed gene are shown in blue. Transcriptional landscapes upstream and downstream relatively to the genomic regions corresponding to G0-10698-*asr* and G0-10703- *yhc*G are shown in Supplementary Figure S5.

Hypothetical ncRNAs inferred from the data of Raghavan et al. (2011) so far remained as the candidates for the experimental validation and in 2019 were even excluded from EcoCyc database (Keseler et al., 2017) as lacking of sufficient experimental evidences of their expression. 5′-specific RNAseq (Thomason et al., 2015; Ettwiller et al., 2016) unambiguously confirmed the transcription of four divergent potential ncRNAs—G0-10698, G0-10703, G0-10702, and G0-10705 (Figure 6; Supplementary Figure S5). Detected TSS in generals correspond to 5′ ends annotated in RegulonDB and are in perfect agreement with PlatProm predictions. Expression of these RNAs demonstrates clear dependence on growth phase, especially for G0-10698 and G0-10703 activated in stationary phase. G0-10703 RNA may be attributed as antisense transcript for *yhc*G gene with yet uncertain function whose expression follows from all sets of experimental data as well as from computational predictions (Figure 6B; Supplementary Figure S5B). G0-10698 and G0-10702 are conserved among *Enterobacteriaceae* (*E. coli, Shigella*, *Salmonella* sp. HNK130, *Salmonella* sp. S13, and *Enterobacter hormaechei* str. RHBSTW-00218). G0-10703 is found in some strains of *E. coli* and in *Salmonella* sp. HNK130, whereas G0-10705 is more widely spread among *E. coli, Shigella, Salmonella enterica*, *Salmonella* sp. HNK130, *Salmonella* sp. S13, *Enterobacter hormaechei* str. RHBSTW-00218, *Citrobacter rodentium* ICC168, and *Citrobacter* sp. RHB25-C09. Thus, undeservedly rejected divergently transcribed ncRNAs may regain their place in *E. coli* genome annotation.

## Discussion

High-throughput sequencing data provide a truly inexhaustible source for the deduction of new molecular relationships in microbial physiology. Mapping of additional TSSs in upstream regions of annotated ncRNAs not only contributes to our knowledge regarding the diversity of specious RNA produced from the same intergenic region, but also enforces us to reconsider functional attribution of certain ncRNAs. More than a half of divergently transcribed ncRNAs are present as a pair or triplet of collinear products transcribed from different TSSs what is consistent with the phenomenon of promoter duplication or multiplication well known for protein-coding genes (Huerta and Collado-Vides, 2003). Despite the role of RNAP “concentrator” additional promoters may provide optimal transcription processivity for ncRNAs according to the cooperation model proposed in Kim et al., 2019. The promoters of known ncRNAs often occur as a sequential array with separate TSSs providing the accumulation of ncRNAs of different lengths and potentially different specificities. The ratio of the activity of alternative promoters for ncRNA is not a stable characteristic and depends on growth conditions. In the case of *och*5 in mineral medium, all three promoters (P1, P2, and P3) demonstrate comparable activity, whereas in LB stationary phase P2 becomes the most active (Figure 2A; Supplementary Figure S2A). For REP-associated potential ncRNA in *ppc*-*arg*E intergenic region, synergistic effect of individual promoters seems to be rather probable (Figure 5; Supplementary Figure S5) although for *och*5 and *tff* (Figure 2A,C; Supplementary Figures S2A,C) cooperation between promoters for ncRNAs may interfere with transcription in opposite direction. It also cannot be excluded that less active promoters represent residual traces or, on the contrary, novel signs on the way of ncRNA selection. It is consistent with the observation that young ncRNAs usually are expressed with low efficiency if compared to evolutionary fixed ones (Kacharia et al., 2017).

Only 30 ncRNAs of 61 chosen as divergently transcribed examples accounting for their genomic context may be attributed as *bona fide* non-overlapped RNAs. The remaining 31 ncRNAs appeared to have TSS within coding part or 5′UTR of the adjacent gene rendering them as potential antisense regulators. Their expression may be subjected to the collision effects (Callen et al., 2004) resulting in substantial decrease in the level of ncRNA caused by convergent transcription. It is illustrated by the case of *oxy*R-*oxy*S pair when engagement of oxyR P2 promoter reduces the level of OxyS (Figure 4). We intended to outline correlation between transcriptional rate of true divergent ncRNAs and their counter-partners. However, the level of transcription of the opposite genes was often hardly detectable whereas ncRNA was rather abundant (*atl*-*ffs*, *yce*F-*sra*B, Figure 2B,D, *yhc*C-G0-10702, Figure 6C). The number of examples available from RNA-seq data and used here did not allow us to conclude that the proximity of highly expressed divergent ncRNAs with transcriptionally “sluggish” genes is more a rule than an exception. Antagonistic rather than supporting effect of divergent transcription on the activity of model promoter mediated by excessive superhelical tension in intervening region was recently reported (Kim et al., 2019). One cannot exclude that this mechanism is implemented in the pairs “ncRNA/adjacent protein-coding gene” when active transcription of ncRNA suppresses the production of mRNA. The extent of mutual promoter interference should obviously depend on the distance between TSSs located on opposite DNA strands, which in the case of true divergent ncRNAs varies in the range from 7 to 290 bp with a median value of 84 bp.

One of the noteworthy observations is that two essential ncRNAs expressed from strong single promoters, Ffs and SraB, appeared to be longer and shorter correspondently than it was previously assumed. The exact nucleotide sequence of RNA, accounting for the position of TSS, is important for the formation of its secondary structure, which is recognized by target molecules. For Ffs, addition of stem-loop structure at 5′end may serve a hallmark for enzymatic degradation. It is confirmed by the finding that Ffs-derivative protruding upstream from the annotated TSP was detected in intracellular RNA fraction indicating the presence of the extended precursor (Alikina et al., 2018). SraB presumed to start from the position 1,146,589 on plus strand (NC_000913.3) that overlaps with early translated region of *yce*F and is likely to be initiated 60 bp downstream. The refined position of TSS was reproduced for all data sets used in our analysis and was also supported by computational prediction (Figure 2D). The exact length of SraB was discussed when it was detected for the first time and the presence of shortened 105 bp product was also mentioned (Argaman et al., 2001). At least seven ncRNAs are likely to wait for rescaling of their size and re-drawing folding pattern. Focusing here on divergently encoded *E. coli* ncRNAs, we did not focus on collinear regulatory RNAs, for which new boundaries can also be established and, accordingly, new functions and ways for the regulation of their expression can be proposed.

## Methods

Raw data available from Thomason et al. (2015) and Ettwiller et al. (2016) (Table 1) were used for the mapping of 5′ ends of transcripts on the genome *E. coli* K-12 str. MG1655 (NC_000913.3). Data processing was carried out in the following stages: 1) 3′-terminal adapter sequence (AAAAAAAAAA–in Thomason et al., 2015; AGATCGGAAGA–in Ettwiller et al., 2016) was cut off; 2) sequences less than 16 bp in length were removed from the whole massive of reads, and residual reads were mapped using bowtie2, generating sam-file1 (Langmead and Salzberg, 2012), with parameter L 16; 3) resulting sam-file1 was used for the extraction of reads perfectly matching the genome (only those containing both labels XM:i:0 and NM:i:0 were selected) generating sam-file2; 4) sam-file2 was converted into bam-format using SAMtools (Li et al., 2009), and the same program was used for the sorting of bam-files; 5) distribution of 5′ ends along both DNA strands was obtained using BEDtools (Quinlan and Hall, 2010). The number of 5′ ends for given position in the genome was normalized using coefficient calculated as ratio of the average number of perfectly matching reads in a series of experiments (N_av_) to the number of perfectly matching reads in given experiment (N_i_). The data shown in Figure 2 (2 S), Figure 4 (4 S), Figure 5, Figure 6 (5 S), and Supplementary Figure S1 represent an average number of normalized counts corresponding to two biological replicates of Ettwiller et al. (2016), two biological replicates for LB OD_600_ = 0.4 (Thomason et al., 2015), 4 biological replicates for LB OD_600_ = 2.0 (Thomason et al., 2015), and two biological replicates for M63 OD_600_ = 0.4 (Thomason et al., 2015). Accession numbers of data used in this study are given in Table 1.

**TABLE 1:** Data used for TSSs mapping.

Accession numbers	Total number of reads after adapter trimming and sifting out of reads less than 16 bp in length	Number of reads perfectly matching the genome	Normalizing coefficient (N_av_/N_i_)
Ettwiller et al., 2016 (M9, mid-log phase)			
ERR930221	14.063,469	11,015,459	0.7322
ERR930222	9,184,197	5,116,134	1.5765
Thomason et al., 2015 (LB 0.4)			
SRR1173969	6,145,538	4886330	0.9927
SRR1173970	5,834,605	4,814,550	1.0075
Thomason et al., 2015 (LB 2.0)			
SRR1173974	5,627,281	3,341,088	1.3391
SRR1173978	6,967,456	5,072,554	0.8821
SRR1173979	7,691,759	5,861,466	0.7633
SRR1173980	6,214,631	3,621,841	1.2353
Thomason et al., 2015 (M63 0.4)			
SRR1173985	8,368,755	5,128,824	1.0292
SRR1173986	8,445,228	5,428,622	0.9724

## Data Availability

Publicly available datasets (from Thomason et al., 2015; Etwiller et al., 2016) were analyzed in this study. This data can be found in European Nucleotide Archive (ERR930221, ERR930222) and NCBI Sequence Read Archive (SRR1173969, SRR1173970, SRR1173974, SRR1173978, SRR1173979, SRR1173980, SRR1173985, SRR1173986).
