# Supplementary material for: Divergently Transcribed ncRNAs in Escherichia coli: Refinement of the Transcription Starts Assumes Functional Diversification
Source: Front Mol Biosci. 2021 Mar 3;8:610453. doi: 10.3389/fmolb.2021.610453 (PMC7967276; doi:10.3389/fmolb.2021.610453)
Supplement: Supplementary file 2 [file table2.docx]

**List of REP-containing genomic regions with potential to produce divergentely transcribed sRNAs**

Color designations: Initiation codon of the one of the pair of ajuscent genes is marked by green color, termination codon of the another gene is yellow, REP elements are marked by grey, TSS related to divergent sRNA – red, TSS of protein coding gene – blue, potential intrinsic terminator – cyan. The qualitative assessment of the expression level is mentioned for putative ncRNA (“low” - 3-10 reads; “moderate” 10÷100 reads; “high” - more than 100 reads for data sets Thomason et al., 2015 (LB 2.0).

1. yidB-gyrB (REP + strand, promoter + strand, considerable expression) 4e-13

3877441 cgacaacttc atcaaaaagc cccatgataa tcacctgtaa agcgttacgt gttgacccaa

3877501 aaagtataga tttgcgggat gataattgcg gattgcagaa ataaaaaggg cggagatgat

3877561 ctccgccctt ttcttatagc ttcttgccgg atgcggcgtg aacgccttat ccggcctaca

3877621 aaatcatgaa aattcaatac attgcaagat tttcgtaggc ctgataagcg tagcgcatca

3877681 ggcacgctcg catggttagc gccattaaat atcgatattc gccgctttca gggcgttctc

3877741 ttcaataaac gcacggcgcg gttcaacggc gtcgcccatc agcgtggtga acaactggtc

3877801 ggcagcaatc gcatctttaa cggtaacgcg cagcatacga cgactttccg ggtccatagt

3877861 ggtttcccac agctgttccg ggttcatctc gcccagacct ttataacgct ggatggagag

1. pgi-yjbE (REP - strand, promoter – strand, considerable expression) 4e-13

4235341 agaaatcagc agccacgata gctcgaccaa tggtctgatt aaccgctata aagcgtggcg

4235401 cggttaatca tcgtcgatat gtaggccgga taaggcgttc acgccgcatc cggcaaccga

4235461 tgcctgatgc gacgcggtcg cgtcttatca ggcctacagg tcgatgccga tatgtacatc

4235521 gtattcggca attaatacat agcacgattg attaaataac cttaataaca atgccgacgt

4235581 tatgtcggca tttttttatc agataaatcc ccttgtctgt aatttaacgg aaatcatacc

4235641 gtgaggttaa tcctaaaata gatttttaat cgttgtttat ttcggaaaat acgcagatta

4235701 attgcttttg tttttatttt aagtttatga tttttattgt tatttaaata taagttgaaa

4235761 cttatatttg atattcattc caattatcct aaaacgccat cgctaattcc ccgcgccgta

4235821 attcgcatgc tttagttgtg tatactcgat cccgcccgaa atgtttttgg gtaaatctcc

4235881 attcattcaa tgaagggaaa ttgttatgaa aaaagttctg tatggcattt ttgccatatc

4235941 tgcgcttgcg gcgacttctg cgtgggctgc acctgtacag gtgggcgaag cggcagggtc

3. ybiO-glnQ (REP + strand, promoter + strand, low expression) 9e-11

845401 gtggttgtaa cgccgggtat ggataccgcg tgggcaggtg cgcccagcag gcagaagagg

845461 atgaacagga tccaccgcat gactcctcca gtgagaaaat agagcaaaaa gataagtata

845521 gatgctggag gaggggcgat tttaaatgag aggaatctgg tgtgcctccc tttcgggtga

845581 aagggaggaa gggatattaa gcaggttgct gacaacgtgc gggctttttt gccggatgcg

845641 gcgtgaacgc cttatccgtc ctacaagttc atgcaatttt aataagttgc cagatatcct

845701 gtaggcctga taagcgtagc gcatcaggca atgtgtctta ttaagagacg tgctgcaaaa

845761 attcctgcaa gcgctggctc ggcgggttct tgatcaacac ctgcggattg ccatcttccg

845821 caatccggcc tttgtcgata aagatcagcc gcgaagctac tttctcggca aaaccgattt

845881 cgtgggtcac gatcaccatc gtcatccctt cttcagccag atcctgcata accttcagca

4. efeB-phoH (REP - strand, promoter - strand, considerable expression) 9e-11

1084561 aaacctatcg gcggcggtta tttttttgcg ctgccggggg tgaaggacgc gaacgattat

1084621 ttcggaagcg cgttattgcg ggtttaatgt ttttaggcgg ataaggcatt tgtgcgcaga

1084681 tgcctgatgc gacgcttgcg cgtcttatca tgcctacaat cagtgcgggt ttggtaggct

1084741 ggataaggcg ttcacgccgc atccggcgat cgtgcactga tgcctgatgc aaatcctgct

1084801 gaaagcacac agcttttttc atcactgtca tcactctgtc atctttccag tagaaactaa

1084861 tgtcactgaa atggtgtttt atagttaaat ataagtaaat atattgttgc aataaatgcg

1084921 agatctgttg tacttattaa gtagcagcgg aagttcccgg cagtgatagt cagtcactat

1084981 ggagatcgcg gatggtaacg tcctgtactg gacatgtttt agataatcaa cgcgccacca

1085041 ctcgcggagt tttctcctcc ggtagtcatc tcgtcacttt gcattttcaa cctcatcctt

5. malF-malE (REP – strand, Promoter + strand, considerable expression) 9e-11

4245001 caggccgagc agacctagca ctgaccattt cagcgcgtcg ctttgccacc aatgtttctt

4245061 tttaatgaca tccatggggt tcttcctcat tccaggacgg ataaggcttt cacgccttat

4245121 ccgacaacaa ctgcctgatg cgacgctgac gcgtcttatc aggcctacat acgtttcggt

4245181 tttgtaggcc ggacaaggcg ttcacgccgc atccggcatt tcacagcatt acttggtgat

4245241 acgagtctgc gcgtctttca gggcttcatc gacagtctga cgaccgctgg cggcgttgat

4245301 caccgcagta cgcacggcat accagaaagc ggacatctgc gggatgttcg gcatgatttc

4245361 acctttctgg gcgttttcca tggtggcggc aatacgtgga tctttcgcca actcttcctc

4245421 gtaagacttc agcgctacgg cacccagcgg tttgtcttta ttaaccgctt ccagaccttc

6. lamB-malM (REP – strand, Promoter – strand, moderate expression) 9e-11

4249261 ggcgacagcg acgagtggac cttcggtgcc cagatggaaa tctggtggta atagcaaaac

4249321 ctgggccgga taaggcgttt acgccgcatt cggcaaccaa cgcctgatgc gacgcttgcg

4249381 cgtcttatca ggcctacaac ggctgtcaaa tgtaggccgg ataaggcgtt tacgccgcat

4249441 ccggcataaa aacaggttgt cattatctga aaggggcgaa agcccctctg attatcgggt

4249501 ttagcgcgct attgcctggc taccgctgag ctccagattt tgaggtgaaa acaatgaaaa

4249561 tgaataaaag tctcatcgtc ctctgtttat cagcagggtt actggcaagc gcgcctggaa

4249621 ttagccttgc cgatgttaac tacgtaccgc aaaacaccag cgacgcgcca gccattccat

7. iscR-trmJ (REP + strand 3 promoters + strand; moderate expression) 4e-10

2661961 gctggaaacc agaccatttt tacgcagacg ggaaaacagt tgttccagat aagaaaggga

2662021 aattccctga cgttcggaaa tatcagccaa cggtaccggg cccgcttcag agttgagcgc

2662081 aacgtcaagc attgcggtca cggcatagcg ccctttagat gtcagtctca tgtcttactt

2662141 cacctcaaac tcgcccctgc ccggggtttt ttattgtaaa agtgggggta ttgcatagca

2662201 gggtcaagtc tgacattccc gagtaaattg gtcaactatt tacttgactg atttagtcgg

2662261 gtatttaacc ttcagtgcca tttttttatc gtggcgtttg tacctgttgt cggatgcggc

2662321 gtgaacgcca tatccgccct ggggttctgt acactgtagg cctgataaga cgcattacgc

2662381 gtcgcatcag gcaacggctg tcggatgcgg cgtgaacgcc ttatccgacc tacggttctg

2662441 ttcactgtag gcctgataag acgcattacg cgtcgcatca ggcaacggct gtcggatgcg

2662501 gcgtgaacgc cttatccgac ctacggttct gttcactgta ggcctgataa gacgcattac

2662561 gcgtcgcatc aggcaacggc tgttattcgg ccttgttacc tttattctgc tgctcaatag

2662621 aagccagaat cccgcgcagg atattcaact cctggctttc cgggcgcgca cgggtaaaca

2662681 gacggcgcaa tttattcatc acctgccccg gatggttttc acggataaaa ccggttgcca

8. ytfQ- ytfR (REP –strand, promoter – strand; low expression) 1e-09

4449901 tctatagttt tttcgcgaac atcttttaac caataataac taccccgacg aggacaaccc

4449961 tatgtggaaa cgcttactta tagtctctgc agtctcggca gccatgtcgt ctatggcgtt

4450021 ggccgctcca ttaaccgttg gattttcgca ggtcggatcg gaatcaggct ggcgtgccgc

4450081 agaaaccaat gtggcgaaaa gtgaagccga aaagcgcgga atcacgttga aaattgccga

4450141 tggtcagcaa aagcaggaaa accagattaa agcggtacgt tccttcgttg cacaaggggt

4450201 ggatgcgatc tttatcgctc cggtggtcgc gacaggttgg gaaccggtat taaaagaggc

4450261 gaaagatgcc gaaatcccgg tattcttgct cgatcgttcc attgatgtga aagacaaatc

4450321 tctctatatg accaccgtca ctgccgacaa catcctcgaa ggcaagttga ttggtgactg

4450381 gctggtaaaa gaagtgaatg gcaaaccatg caacgtggtg gagctgcagg gcaccgttgg

4450441 ggccagcgtc gccattgacc gtaagaaagg ctttgccgaa gccattaaga atgcgccaaa

4450501 tatcaaaatc atccgctcgc agtcaggtga cttcacccgc agtaaaggca aagaagtcat

4450561 ggagagcttt atcaaagcgg aaaacaacgg caaaaacatc tgcatggttt acgcccataa

4450621 cgacgacatg gtgattggtg caattcaggc aattaaagaa gcgggcctga aaccgggcaa

4450681 agatatcctc acgggttcca ttgacggtgt accggacatc tacaaagcga tgatggatgg

4450741 cgaagcgaac gccagtgttg aactgacgcc gaatatggca ggtcccgcct tcgacgcgct

4450801 ggagaaatac aaaaaagacg gcaccatgcc tgaaaagctg acgttaacca aatccaccct

4450861 ttacctgcct gataccgcaa aagaagaatt agagaagaag aaaaatatgg ggtattgagg

4450921 gttgctatgc ctgatgccga ttcgtaggcc ggataaggcg ctcgcgccgc atccggcgat

4450981 ggtgcactga agcctgatgc gacgcttacc gcgtcttatc atgcctactg ggagcacgct

4451041 ttacaccggg ggaaaccatg acgaccgacc aacaccagga gatcctccgc accgaaggat

4451101 taagtaaatt tttccccggc gtcaaagcgt tagacaacgt tgatttcagc ctgcgccgtg

4451161 gcgaaatcat ggcgctgctc ggtgaaaacg gggcgggaaa atcaacgcta atcaaagcat

9. ppc-argE (REP +strand, promoter + strand; high expression) 6e-09÷2e-08

4152961 gcgtgaagat ttcgacaact tacggatagt ttctacgcgt tcaagaatgt gttctcccaa

4153021 cgcatccttg atggtttctc ccagcacttt gccgagcata ctgacattac tacgcaatgc

4153081 ggaatattgt tcgttcatat taccccagac accccatctt atcgtttgat agccctgtat

4153141 ccttcacgtc gcattggcgc gaatatgctc gggctttgct tttcgtcgtc ttttataaag

4153201 ccacgtaaaa gcggtgacgt caaatgctgc gaaatcgctt cagcaaacga ataaatagca

4153261 ggaatttacg tcattaaatt cacgacgctt taaataagcg taacttatgg aaatgttaaa

4153321 aaatcgcccc aagtaacacc aaaggtgtag gtcggataag atgcgcaagt atcgcatccg

4153381 acattattgc ggcactggag tttggcaaca gtgccggatg cggcgcgagc gccttatccg

4153441 gcctacagtt gggcatcgtt tgagtcactg tcggtcggat aagatgcgca agtatcgcat

4153501 ccgacattat tgcggcactg gagtttggca acagtgccgg atgcggcgcg agcgccttat

4153561 ccggcctacg gttgggcatc gtttgagtca ctgtaggtcg gataagatgc gcaagcatcg

4153621 catccgacat tattgcggca ctggagtttg gcaacagcgc cggatgcggc gcgagcgcct

4153681 tatccggcct acgttttaat gccagcaaaa atggtgaatt acctgggtta tcagttcgcg

4153741 ggtgggcttg ataaaccgtg tttccagata ttcatcaggt tgatgagcct gattaattga

10.gmhA- yafJ (REP + strand, promoter + strand, codirected transcribed REP; low expression ) 9e-08

244021 ttcgcgtacc gcactttggt tatgccgacc gcattcagga gattcacatt aaagtgatcc

244081 atatcctgat ccagttgatt gaaaaagaga tggttaagta agtctggcgt aggccggata

244141 agacgtttac gccgcatccg gcatttgtgc gctgatgcct gatgcgacgc tgacgcgtct

244201 tatcatgcct acaaatctgt acgcgaaccg taggccgaat aatgcgttca cgccgcatcc

244261 gacctgaaaa ttcttaaatc aatcttcgcc gggggccatg cgctcccgct gttgtggagg

244321 ttacccatgt gcgaattgct cgggatgagc gccaacgtcc ctaccgatat ctgctttagt

244381 ttcaccgggc ttgtacagcg tggtggtgga accgggccac ataaagatgg ctggggcatt

244441 accttttacg aaggtaaagg ctgtcgcaca tttaaagatc cacaacccag ctttaattcc

11.yhgF-feoA (REP – strand, promoter – strand; high expression) 4e-07

3539581 ggcggcggta atgaacgccc gcaaaacaac cgcccggcag ccaaaccacg cggtcgtgaa

3539641 gcgcagcctg ccggtaatag cgcgatgatg gatgcgctgg cggcggcaat gggcaaaaaa

3539701 cgttaaacgc ccgtactggc ctacggttcg aatttgcacg aaatcgtagg gcagataagg

3539761 cgttcacgcc gcatctggca acgaacgcct tgcctgacat aaaagtgccg gagaatatct

3539821 ccggcatttt tattccacag ccaaactcat aatatattcc ggcaatattt atcatttcat

3539881 taacaactga aaccttaatt aaacattagc cagtccgggt aattcactat tcgaattata

3539941 ttttcgctgc gatataacct tgagccacat caacattgag tcagattatt attcaaacca

3540001 acattcgcac acattttaag tattgctgat agaaaccatt ctcattatca ttgtgttgtt

3540061 gattatttaa tctctccttt gttggcaaat catctggtct catgtcgctg tcaaacgccc

3540121 catgaggtag ttatccagtt aatgagaaac aagtaggcac ctatgcaata cactccagat

3540181 actgcgtgga aaatcactgg cttttcccgt gaaatcagcc cggcatatcg ccaaaaactg

12. gatY-fbaB (REP + strand, promoter + strand; moderate expression) 1e-06

2177041 gtaaatgtgc caggcgttcc ggcgatgatg accggcgcat gcaggttggc agcggtttct

2177101 accaccactt gcatcgtttc gagattgtga atattgaatg ccggaaccgc ataaccgccg

2177161 cgctgtgcgt tgttcagcat ctgctttgtc gataccacgt acattttcat atcctgtcgt

2177221 ttgttttcga tttcaaaata taatgaaatt atttgttttt aaatatcgag ataacgatca

2177281 caaaaacgac aatatgaaaa ttattcgagg agtgaaaggc aaaaaaacgg cctcccgata

2177341 gggaagccgt agcaaagtgc gcgtgttttt atgccggatg cggtgtaaac atacgtggca

2177401 gtgctatgta ggcatgataa gacgcttcag cgtcgcatca ggcataggtt gccggatgca

2177461 gcgtaaacgc cttatccgtc ctacggggtg gtgttgttcg tataaggcgt atcaggcgat

2177521 agtaattttg ctatcgagat aaacgtcctg cacggcgtta atcagtttca cgccgtcagc

2177581 catcgatttc ttgaacgctt tacgtccaag aatcagcccc attccgcctg cgcgtttgtt

13. ycfP-ndh (REP – strand, promoter – strand; moderate expression) 6e-06

1165561 gccagcggac atctgaagag ttgcatcatt attacgagat tgtctgggac gaagagcaga

1165621 cgcacaaatt caagaatatc tccccgcatt tacagcgcat taaagcgttc aaaaccctcg

1165681 ggtaaatgcc ctcgtcgcat caggtaacct tgccggtacc tgatgcgctc cgaattctgt

1165741 gggtcggata aggcgtccac gccgcatccg acagtcgagc atcaatgcct gatgcgcttc

1165801 ttatcaggcc taccgaacgc cctgcataca cccctcactc tatatcactc tcacaaattc

1165861 gctcaaataa taaacaataa actctgtttt ttgatctcac ccggtaaagt cgcctatctt

1165921 ttcagcaaca aaacttgatt aacatcaatt ttggtatgac caatgcacca ttcatgttat

1165981 tctcaatagc gaagaacatt ttcattgctg taacctgttg ttaattaaga gctatgttaa

1166041 taaccattaa ttaacaattg gttaataaat ttaagggggt cacgttgact acgccattga

1166101 aaaagattgt gattgtcggc ggcggtgctg gtgggctgga aatggcaaca cagctggggc

14. nepI-yicN (REP + strand, promoter + strand; TSS on – strand not detected; moderate expression) 6e-06

3841621 acgggcaaaa actcaacgat aatcagacag gcgacacaaa acgccaccga gaaaacggct

3841681 gaccagttcg gtcgggtgat ggcatccgcg ccgcggtttt cggcaataaa ttcactcatg

3841741 gtgttacccg tggcatgttt caggggaaaa gccgaccact ttaacattga aatgtgtgac

3841801 gcatttaacg tttttgcaac ttttacggtg ttgcttgagc tggagttaag atcgaaacgg

3841861 aacagggcaa agattaccgg atgcggcgtg aacgtcttat ccacccgaca ggtcaggcgc

3841921 ggtctgtagg ctgataagac gaaacggttt caatgcaacc agtgaacgcc ctcggcaggc

3841981 tgaaagaacg cgttatagcg catctgaaaa gcattaatat cctgcatttc aggttcggtt

3842041 tcgcgcagaa aacctagcgc cagcctgacc tgagcgtcgg taatcggcgc ggcgaggcga

15. yijO-eptC (REP + strand promoter + strand; high expression) 6e-06

4148101 agccaccagc aggaacgtac agcacatcgc cgggaactaa cgttgctcca gcgccggtat

4148161 caacaaactc accttccagc acaatttcca gccgtggaaa atcgacctga tacgcaagat

4148221 cgggcaccgg gccgttactg ctggcaaagt aaatctggcg cagggacagc gggccgttga

4148281 tcaggcggga gagcagatag ctgacgtcgt gatacatgtt attcccttag gtatgtagac

4148341 gggaaaacag taactggcat tattaaaaaa ggccacctga aaagtgtggc ctgaggggag

4148401 ttcgatgtaa acattgcggc agttgtattg ccggatgtgg cgtaaacgcc ttatccagcc

4148461 taccgccgcg atctgtaggc cggataagac gcgtcaagcg tcgcatccgg caaatagcgc

4148521 ctggctgata attactgatt acccacctga tcgccatagg gcagtgtgtc gtaatcgatc

4148581 agtgcgtttt tcttatacgg gttaccaatc cagcgggtag tttctttgaa ctgcggattc

16. yhfZ-trpS (REP – strand promoter + strand; moderate expression) 4e-13

3512221 atgaacggca ttcatttgcc agctcatcta tggttttcaa tcgattaccg cacttttcac

3512281 ccaacaaata acgggccagc gtcgtgatga cgacgccttc ttttttgata aacgttcgac

3512341 gcataataaa ttttcagtaa attgaatatt tatatcttca ggaatttgaa gataaggcgc

3512401 aacagcgaga tgtggaaacg gcgaggcact tcacattttt tcggattatt gcaaatgagg

3512461 ataaaaaaac cgggtttccc cggtttcaga gtgatgataa aagcaaaatt gcctgatgcg

3512521 ctacgcttat caggcctaca tttccttgca atatgtgcat tactttgtag gccggataag

3512581 gcgttcacgc cgcatccggc atgaacaaag cgcaatttgc cagcaatagt gaattacggc

3512641 ttcgccacaa aaccaatcgc ttcgtacacc gcttttagcg tacgggaagc gtgcgcgctg

3512701 gctttttccg cgccatcttt catcacctgt tgcaggaagg cttcatcgtt gcggaaacgg

17. yiiX-metJ (REP + strand promoter + strand; low expression ) 4e-13

4127761 gtatgggtcg ccagttggat cgctttactt tgcgatgagc gagagatctg aaagatgatg

4127821 tcgccggttt gtggctgcca ggcaaaggca ggtacagaaa ccagcaggct gaggatcagc

4127881 agcctgtttt tcatagttaa acgtccatgt ataaaaagcg gtgggtcgca gacaacgtgc

4127941 tcgttgttta tgccggatgc ggcgtgaacg ccttatccgg cctacaagtt cgtgcaaatt

4128001 caataaattg caatatgacg taggcctgat aagcgtagcg catcaggcga ttccactccg

4128061 cgccgctctt ttttgcttta gtattcccac gtctccgggt taatccccat ctcacgcatg

4128121 atctcttttg ccgcttccgg gatttcgtcg ctgcgctctt tacgcagatc ggcatcatcc

4128181 ggcaaaggtt gcccggtaaa ggcatgcaga aacgcttcgc acagcagctc gctgttggta

18. aceA-aceK (REP – strand Promoter – strand, REP-containing putative RNA – antisense to aceK 5’UTR; moderate expression) 4e-13

4218301 ctcaccagca ggaagtgggt acaggttact tcgataaagt gacgactatt attcagggcg

4218361 gcacgtcttc agtcaccgcg ctgaccggct ccactgaaga atcgcagttc taagcaacaa

4218421 caaccgttgc tgactgtagg ccggataagg cgttcacgcc gcatccggca atcggtgcac

4218481 gatgcctgat gcgacgcttg cgcgtcttat catgcctaca gccgttgccg aacgtaggct

4218541 ggataaggcg tttacgccgc atccggcaat tctctgctcc tgatgagggc gctaaatgcc

4218601 gcgtggcctg gaattattga ttgctcaaac cattttgcaa ggcttcgatg ctcagtatgg

4218661 tcgattcctc gaagtgacct ccggtgcgca gcagcgtttc gaacaggccg actggcatgc

4218721 tgtccagcag gcgatgaaaa accgtatcca tctttacgat catcacgttg gtctggtcgt

4218781 ggagcaactg cgctgcatta ctaacggcca aagtacggac gcggcatttt tactacgtgt

4218841 taaagagcat tacacccggc tgttgccgga ttacccgcgc ttcgagattg cggagagctt

19. yiaW-aldB (REP + strand, promoter + strand; high expression from distal promoter, low activity of REP proximal promoter; TSS for yiaW was not detected) 2e-11

3754321 acgttttttg gcaatcaggt agggaatatc atgtaaaata atgatcccat agaagatcac

3754381 cagaaataca aaaataagca ctcccagtgc aaaatagtcc aggaacatat ttccctcaaa

3754441 gaatataaaa aagaacaatt aacgcatatt atgaaatgcc atgatgcagt gcaatttcct

3754501 tatttatata acgacatatt gttttttata tactttctta aatgtgctgt ctggtttttc

3754561 aacaggacga ggtttatcat attgaaatag tggaactatt ggccaaacta atgaataact

3754621 ccagtaaaac atccacagta cagattaagc gtattaaacc ttcaattatc taccgtttat

3754681 tgctgattgg cctcggatca ccaatggtga tttacggcct ggttcgcccg ctcaccatcg

3754741 aaacgcgaga ttaaactcct gacaaaacac gccccagaaa cgtacttctg ttggcgtagt

3754801 ctgggttatt gcgcacgtag gtttaaaacg taaattgtaa caacgtgcgc tttgtttatg

3754861 ccggatgcgg cgtgtacgcc ttatccggcc tacggtcagg ttcccgtagg catgataaga

3754921 cgcgtaagcg tcgcatcagg caatgaatac ccaatgcgac cagcttctta tatcagaaca

3754981 gccccaacgg tttatccgag tagctcacca gcaggcactt ggtttgctgg taatgctcca

20. sseB-pepB (REP – strand Promoter + strand; low expression, TSS for sseB was not detected)9e-11

2654821 ccaggcaccc agacggtgga ttccagtaga gtacggaaaa aggccgggcg gtgcgccggt

2654881 tcagttgctg ctttttccag caggtcttca agttcgtttt ttgtttcgga cataagaacc

2654941 acaattcatt caacgttcgg ggcgcaaatg cctgatgcgc tacgcttatc aggcctacaa

2655001 gggattcgca atttgttgaa tttgcagaat ttgtaggccg gataaggcgt ttacgccgca

2655061 tccggcatat tagtttacgc cgttaacaga ttagctatcg tgcgcacacc aagtcccgta

2655121 gcgcccgcag accactgttc aaccggcgct ttacggtaag tcgccgagca gtcgatatgc

2655181 agccagcctt gctgatagtt ctcaacaaag tgcgacagga agcccgccgc cgtgctcgcg

2655241 cctgccggat acgccgcgct tccggtattg ttcagttcgg caaagttaga cggcagctgg

21. yjcH-acs (REP –strand Promoter + strand; moderate expression) 9e-11

4285081 cgatcagtaa aataaagccg atataaactg ccagcataat aatcgacagg atggtggcaa

4285141 accgttgccg tttttcgact aactccctga aatgcgcatt gtcttctatc cgctgataaa

4285201 tagtgccatt catcacagat tctccagagg taatgtaggg attgttttaa ttcccgctcc

4285261 cttatgggag aaggttaacg ctcgggtaac ccttgccgaa tgtaggccgg ataaggcgtt

4285321 tacgccgcat ccggcaatca atgcctgatg cgacgctgtc gcgtcttatc aggcctacaa

4285381 accgttaccg actcgcatcg ggcaattgtg ggttacgatg gcatcgcgat agcctgcttc

4285441 tcttcaagca gcttctcgac tacgccagga tcggcaagcg tcgaggtatc gcccaggttg

4285501 ctggtatcgc ccgccgcaat tttgcgcaga atacggcgca taattttgcc ggagcgggtt

22. eutH- eutG (REP + strand promoter + strand, low expression)

2568001 ttaccgagga aacgagcaga accgccgaac tgcgacagga tcctgtctac ggcagctatc

2568061 agcataaaga acatcatgat gtacatgatg atttcgttaa ttcccatcgc ctttactccc

2568121 tgttagttgt tatttattgg cggatgcggc gtaaacgcct tatccgccct acatgtgcaa

2568181 tcccgtaggc tggataagac gcggcaagcg tcgcatccgg caattgcacc gcgccactgg

2568241 cggatgcggc gtgaacgcct tatccgccct acatgtgcaa tcccgtaggc tggataagat

2568301 gcggcaagcg tcgcatccgg catttattgc gccgctgcgt acaggccgac aatctgctcc

2568361 aggctggcgg tacgcgggtt actgcgcaga caaatatctt ccagcgcggc ctgcgcccat

2568421 gcgccgtaat gcgcagatgt cgcaccaaca tcgcccagtc gtttaccaat cccaacttcc

2568481 gcaatcagct cacttaccgc gttaatagcg tcacgatcgt cggatttttt agttcgcagt

2568541 gcccgaccaa tctgactaaa gcgttcacga caaaccatcc ggttaaattc catcaccgtt

23. pppA- sslE (REP + strand, Promoter + strand, low expression)

3114241 tgattgggta acgccaaatc accacattca aaaaactgcc gatgatcaat cctccgacgg

3114301 ttgccaggac gggcatcgcc gtggggtatt gctgaaaaac atcaaaaagc atggttaaag

3114361 gttgtttgtt gtaacttgct ggatgcggcg taaaacgcct tatccgtcct acgggtgtct

3114421 gccagcgcaa ataatcgcgg ctttccccgt ctgtaggccc gataagcagg cgcatcgggc

3114481 aaatgtgtta acccggtgcg ccttatttca tgccggatgc ggcgcgagcg ccttatccgg

3114541 cctacgggct tactcggcag acatcttatg ctcggtaacc tgattaatgg tttccggtcc

3114601 ctgttccggt ttcggcagat cgagtgacgc gagcgtgttg taagccgact ggctcacacc

3114661 gccctcgaag ctcatctcgc tcgcccccgg cagctggtaa gcattcgcgc ccggattcca

24. tldD- yhdP (4 REPs + strand, promoter + strand, moderate expression)

3391921 gccataatca aggcgacgtt cggccagttg accgaggatc gcgaacaagt cctgatgttt

3391981 caggccgttc gccgctagca attgttcact taccaggtta agactcatcg tttttgctac

3392041 tcgttagtta ctgcagtaga ggatttttta cggctgccgg atgcggcgtg aacgccctat

3392101 ccagcctacg gttatgttcc ggtttgtagg cctgataaga cgcacagcgt cgcatcaggc

3392161 aacggctgtc ggatgcggcg taaacgcctt atccgaccta cggttatgtt ccgtttgtag

3392221 gcctgataag acgcacagcg tcgcatcagg caacggctgc cggatgcggc gtaaacgcct

3392281 tatccgacct acggttatgt tccgtttgta ggcctgataa gacgcacagc gtcgcatcag

3392341 gcaacggctg ccggatgcgg cgtgaacgcc ctatccgacc tacggttatg ttctggctcg

3392401 ttggcctttg gcaacgatta tcctatgaga ttggggcaat tacgcgccct cgtcaaatca

3392461 ttgcgctttt tctttacgcg gttggcgcaa cacttcgttg atttgcggat cgtccagcgg

3392521 acccgaaatg tgatagcgca aaatggagac tttgctccac agcggcccca gcactttact

25. rhaD- rhaL (2 REPs – strand, Promoter + strand, moderate expression)

4094101 gcgggatata gcgcggttgt tggtggaaat tgtcgtgata tggtgcgata tcggcgtcat

4094161 ccaggcgtag cgtcaggttg ccgccgttgc gctcatccca gcctttcagc caggcgtcgg

4094221 tggtggcttt gatcattccc tggacaaacc aggactgagt aatgttttgc atgttctgtg

4094281 ttcctgtaaa ttcggtgttg tcggatgcac gacccgtagg ccggataagg cgctcgcgcc

4094341 gcatccggca atcaatgcct gatgcgacgc tgtcgcgtct tatcaggcct acaactattg

4094401 ccgacctgta ggcctgataa ggcacttgtg ccgcatccgg caatcaatgc ctgatgcgac

4094461 gctgtcgcgt cttatcaggc ctacaactgt tgccgacctg taggccggat aaggcacttg

4094521 tgccgcatcc ggcaatcaat gcctgatgcg acgctgtcgc gtcttatcag gcctacaact

4094581 attgccgacc tgtaggccgg ataaggcact tgtgccgcat ccggcaatca atgcctgatg

4094641 cgacgctgtc gcgtcttatc aggcctacaa ctgttgccga cccgtaggcc ggataaggcg

4094701 ctcgcgccgc atccggcagt gtttacccgc ggcgactcaa aatttctttc tcataagccc

4094761 gcacgctctc cagccattcg ctacctgctg gcgtatcgtg acgttggcaa tacatttccc

4094821 agaccgcctg ccacggcaac gatttctgct cttccagcag tgccagacgc gcagtgtaat

26. yafL-rayT (Rep – strand Promoter – strand, low expression)

247321 ggcgtgtatt tgggcgatgg gcaatttatc gagtcgccac gtaccggcga aaccattcgg

247381 ataagccgat tagccgaacc tttctggcag gaccattttt tgggcgcgcg caggattttg

247441 acggaagaga cgattttgta ggacggataa ggcgtttacg ccgcatccgg cagttgtacg

247501 caggtgcctg atgcgacgct ggcgcgtctt atcatgccta cgagcccgcg aatatttgcg

247561 agccgctttc ccgatataaa acaacctcat tgccaacctt tccttttctt cttaccgttg

247621 agaaaaagga gtcgccatgt ctgaatatcg tcgttattac atcaaggggg gaacatggtt

247681 tttcacggtg aatttacgaa atcgtcgaag ccaacttttg accacccagt accagatgct

247741 ccgtcacgcc attattaaag ttaagcgaga caggcctttt gaaatcaacg cctgggtcgt

247801 tttgccagag catatgcact gtatctggac attacctgaa ggcgatgatg atttttcctc

27. artJ- artM (REP – strand Promoter + strand, moderate expression) Antisense to artJ 5’ UTR

900421 tttgccagat cgatatcaaa gccgacaatc tcattattag cacctataga ttcaaagggt

900481 ggataggtgg ctgaaacgcc aaaattgatt ttctctgcgg cagaagcacc gaaagtaaag

900541 gaagcaagta aagcggcaag aactaacttt ttcatgatgg aactcccgtc tgtcaatctt

900601 atgatttttg gccgtgtctg cggcatggga taacaatgcc atcaagtgaa tttatatgca

900661 ataaacatga ttaaataatt taaatgaaat aaaaaagacg gacaacttag tgggttgtcc

900721 gtcttcatta taagaattta tgcactatgt aggccggata aggcgtcccc gccgcatccg

900781 gcacaggcac cgtgctgatg tctgatgcga cgctggcgcg tcttatcaga cctacaaaac

900841 cccccggcga atgtacgcag ccacattaat ttcgccgttc gaatgccagc gctttgcgct

900901 cgatcagacg catcatcagc gtcagcaggc cgttaacgac caggtaaata atccctgccg

900961 caccgaacac cattacatcg taggtgcgtc cgtacaacaa ctggctgtat cccatcactt

901021 ccatcagcgt aatggtgtat gccagagagg tacttttgaa taccagcacc acttcgttgg

28. osmF-bglX (REP + strand Promoter + strand, moderate expression)

2219281 caccacagga gtcgttccga gttgcacttt attcaccgtc ggtacgccgt ggctttccag

2219341 cacctgcaaa atgatattgc cgagtagcgc accttcggta tcgatttttg aaccgacttt

2219401 aacgggggaa gccgcctgta gcggcaggct cacggctgcc aacataacca gtgaacctgc

2219461 ccagagcttt aagagtggca tgatgctttc ctcattcttt tactgttgtt ttcagcgaat

2219521 taagagaaaa gcatagttga taatggcggg gttagcttga gcggaaggat tcagttgcag

2219581 aatcagataa atacttaaga ggcatattcg gtccggcgtt tcctgccgga tgcggcgcga

2219641 gcgccttatc cggcctacaa agggcgcaaa cgtcgcgccc ttactaaagc attacagcaa

2219701 ctcaaactcg cctttcttaa cgcgtgcgga atcagtgccg ataaagacat tgaacttgcc

2219761 aggctcggcg tcatatttca tctgttgatt ccagaacttc agcgcctcaa tatcgatcgg

2219821 gaagctgaca gtctgagttt cgcccggttt cagggtgatt ttctcaaagc ctttcagctg

29. cdd–sanA (REP + stran, Promoter – strand, low expression) Antisense to sanA early translated part

2232601 attacccgga tatccagcgc gcggttctgg cagaaaaagc cgatgcgccg ttgattcagt

2232661 gggatgccac ctccgcaacg ctgaaagctc tcggctgtca cagtatcgac cgagtgcttc

2232721 tcgcttaagc ctggtgccgg atgcggcgtg aacgccttgt ccggcttgcc agccctctcc

2232781 tggtgtcgaa attcccggca aacagtttgc cgtttcttgc gcaaaaccag cgggtaaagt

2232841 agcctgatgg aaattttcct tagatcgagt ctcctgcatg ttaaagcgcg tgttcctcag

2232901 cctgttagtc ctgatcggct tgctgctgtt gactgtgctc ggcctcgatc gctggatgag

2232961 ctggaaaacc gcgccttata tctacgacga attgcaggat ctcccctacc gccaggtcgg

2233021 tgtggtgctc ggaacagcaa aatattatcg tactggcgta attaatcagt attatcgcta

2233081 ccgcattcaa ggagcgatta atgcctataa cagcggtaag gtaaattatc tattactgag

2233141 cggcgataac gcattgcaaa gttataatga gccgatgacc atgcgcaaag atttaatcgc

30. purC-bamC (REP + strand Promoter + strand, low expression) Antisense to purC 5’ UTR

2597521 gacgtatcat tgcggaattc gagcaccaac aggtccgggt tttccgtgct gtatacggtt

2597581 ttcgctttac cacgatacaa ctcagcttgc ttttgcatct ttatcactcc tgggtgtgaa

2597641 ttaacgtttt aaaatctttt gctgtctggt gtgccggatg ttttgtcgga tgcggcgtga

2597701 acgccttatc cgaactacgt ccgacaaaca aatttcgtgc gaattaccgc taaaatcgcg

2597761 ttttcctgca gacgcacacg tttgcgtatc atatcagaaa aaagggccgg atgattccag

2597821 ccctgtattt ttacttgcta aacgcagcct ggaagacagc taccagcgcg tcgttctgac

2597881 tctgagtcag agtatgacct ttcggatcga tgaactgtag gctgctgcgg ttatctaaat

2597941 cgccaacctg cagtttatag tcaccggatg ccaggcctgg atcgctcgcg cccagttcct

31. ugpB-livF (Rep + strand Promoter + strand, moderate expression)

3592081 ggcgcgttgc cggtacgaaa tgcggcaatc cccgcgctta aattctgttc gtagttgcct

3592141 ttataggtcg gtacaatttt gtaatccggg ttttcggcgt taaaacgttg ggccagagaa

3592201 tccacctctt tacccagttc cccttccata gaatgccaga acggaatggt cgtcactgcc

3592261 tgtgcattcc ccattaacgc cagtccgagc gccagtgctg aagctgtata atgtaacggt

3592321 ttcatcgttt atctctcttg ttgtaccgaa tgcgcgaatt cacgcgtttt atgctcgcgg

3592381 ggtaacatga catgctcgaa ttacagaaaa ataacttttt tgttacattt gtaagatagt

3592441 aaggtgtcag aaagatgaca aggcggtgac ggcgtgggtg agggaaaatg ggagatgggg

3592501 cacggataag cgggaaaata tagaaggtct gaatcaaact ctacagattg ctcatcgttt

3592561 catgccggat gcggcgtaaa cgccttatca ggcctacaag atcgtgcaaa ttcaacatat

3592621 tgccactcac ccagtaggcc tgataagcgc agcgcatcag gcaattttac atttgtcacc

3592681 tgtctcaaag gagtcttttg actccctatc aatcaacgtg ttattacccg cctaaatacg

3592741 cacttctcac cgcttcattc gccagcagcg catcaccagt atcggaaagc actacatggc

32. gabT-gabP (Rep – strand, promoter – strand, moderate expression)

2793781 cgggctgggg gcgatgatcg ccattgagct gtttgaagac ggcgatcaca acaagccgga

2793841 cgccaaactc accgccgaga tcgtggctcg cgcccgcgat aaaggcctga ttcttctctc

2793901 ctgcggcccg tattacaacg tgctgcgcat ccttgtaccg ctcaccattg aagacgctca

2793961 gatccgtcag ggtctggaga tcatcagcca gtgttttgat gaggcgaagc agtagcgccg

2794021 ctcctatgcc ggaggcgacg ctgcgcgtct tgtccggcct acggggatcc aggtcggata

2794081 aggcgtttac gccgcatccg acaatctgta cgtgaacagg aagaaatcta tgttggccgg

2794141 gtaaggcgga gccgctctcc ggcaaaaaga atcaataaca attatacgcg tgacccggcg

2794201 cgggaaatgt cggggcgctc tcccaagtga cacactttcg agaggattca ggatggggca

2794261 atcatcgcaa ccacatgagt taggcggcgg gctgaagtca cgccacgtca ccatgttgtc

2794321 tattgccggt gttatcggcg caagtctgtt tgtcggttcc agcgtcgcca tcgccgaagc

2794381 gggcccggcg gtattactgg cctatctgtt cgccgggcta ctggtggtta tgattatgcg

2794441 gatgttggcg gaaatggcgg ttgccacgcc cgataccggt tcgttttcca cctatgccga

33. metK-galP (Rep – strand, promoter + strand, low expression)

3087661 agaaagtgcc ttctgaacaa ctgaccctgc tggtacgtga gttcttcgac ctgcgcccat

3087721 acggtctgat tcagatgctg gatctgctgc acccgatcta caaagaaacc gcagcatacg

3087781 gtcactttgg tcgtgaacat ttcccgtggg aaaaaaccga caaagcgcag ctgctgcgcg

3087841 atgctgccgg tctgaagtaa tctttcttca cctgcgttca aaggccagcc tcgcgctggc

3087901 ctttttcttt tggataggcg ttcacgccgc atccggcaaa aaaaccgccc gcacaataac

3087961 atcattcttc ctgatcacgt ttcaccgcag attatcatca caactgaaac cgattacacc

3088021 aaccacaaca gacaaagatt tgtaatattt tcatattatt attcggtttt cacagttgtt

3088081 acatttcttt tcagtaaagt cttaattgca gataacagcg tttaatctat gatgatataa

3088141 ctcaattatt ttcatgcact taaatcataa ctaagataaa tgttagtgta agcgattaca

3088201 ctgatgtgat ttgcttcaca tctttttacg tcgtactcac ctatcttaat tcacaataaa

3088261 aaataaccat attggagggc atcatgcctg acgctaaaaa acaggggcgg tcaaacaagg

3088321 caatgacgtt tttcgtctgc ttccttgccg ctctggcggg attactcttt ggcctggata

3088381 tcggtgtaat tgctggcgca ctgccgttta ttgcagatga attccagatt acttcgcaca

3088441 cgcaagaatg ggtcgtaagc tccatgatgt tcggtgcggc agtcggtgcg gtgggcagcg

3088501 gctggctctc ctttaaactc gggcgcaaaa agagcctgat gatcggcgca attttgtttg

34.sapA-ymjA (Rep + strand, promotor + strand; low expression)

1356901 cagcagtcga tcataaaact gggcggcaag ggtatcgaca attaacccac tgctcgcttt

1356961 ggatgggtta aaggtgttga cttgcccgct gacgcaatag acaaaaccgc tgtcgcggat

1357021 atcagcatgc gggggagatt caggcgcggc gattgcctga ccactcacaa gtccagcaat

1357081 caccaaaaga gacgataata cctggcgcat aatattaagg gattttatgt aaagaggcta

1357141 tcttactaat atttaatgac atttgccatt accgtttgtg ttcaggggtc gtaatgaggg

1357201 ccgtgtggtt ggtcgttgaa caggtgacgt cgccatctgt tcaacattcg tacccgtgat

1357261 ttctctataa ctataactca cagaacaact tagcgaggag cagagcggta cgatcgtgag

1357321 aaacccatta aagcacgctg cagtgcgtgt cattgttagc cagatgcggc gtgaacgctt

1357381 tatccggaca acgatactga ccgatcgtct gcaattaaat cattactcat taccccattg

1357441 attcaaaaac tctgcgatct catcaatgcg tacgggatta atccccgctt cagcagccat

1357501 ttcatgttgg gcttcttcgc tgatctcttc attgttcatc aaacgggtga gcagtaactg

1357561 gaagtagtgg gccagcggtg tgcgttcagc ctctgcgaca ggctctgcgc attcggtcgc

1357621 gtactcatcg gcaatatcaa aatattttag cgggatatcg tggttcatta tttgcccctg

35. rbfA-infB (Rep + strand, promotor + strand; TSS on – strand no detected, moderate expression)

3312541 aatgacccga tagcgtttgt cggagtccag cagatactgg gaaaacttcg tcgcttcccc

3312601 gaggcaaatc ggcaacatgc cggtcgccag cgggtccagc gcaccggtat gcccggcacg

3312661 gttggcgtta tatatacgtt tcactttttg cagcgcatcg ttgctggaca taccctgagg

3312721 tttatccagc aacaaaacgc cgttaatgtc gcgaccgcga cgacgaggac gactcattag

3312781 tcctccttgc tgtcgtccgg gttaacacga cgttcttcgt catgtttgac cacgctggtc

3312841 accaggtttg acatgcgcat cccttcaacc agagagttgt cgtagaagaa ggtcagttcc

3312901 ggcacgatac gcaggcgcat cgctttcccc agcaggctgc ggatgaaacc agaagcttct

3312961 tgcaacgctt tgatgcccgc tttaaccgcg tcttcatctt tgtcgttgag gaacgtcaca

3313021 tatactttgg catacgccag gtcgcgagac atttcgacac cggaaacggt ggtcatcatg

3313081 cccaggcgag gatctttaat ttcacgctgc aggatgagag cgatctcttt ttgcatttcc

3313141 tgcgctacgc gctgcgggcg accaaattct ttcgccataa taaattctcc tgacaaaaaa

3313201 ggggctgtta gccccttttt aaaattaatt tcaggtggaa gggctgttca cgttgacctg

3313261 ataagacgcg ccagcgtcac atcaggcaat ccatgccgga tgcagcgtaa acgccttatc

3313321 ccgcatggaa ccctaaaaac cttaagcaat ggtacgttgg atctcgatga tttcgaatac

3313381 ttcgatcaca tcgccagtgc ggacgtcgtt gtagttctta acgccgatac cacattccat

3313441 accgttacgg acttcgttaa cgtcatcttt gaagcggcgc agggactcca gctcgccttc

3313501 gtagataacc acgttgtcac gcagaacgcg gatcgggttg tgacgtttaa ccacaccttc

3313561 ggtaaccata cagcctgcga tggcaccaaa tttcggcgat ttgaacacgt cacgaacttc

3313621 cgccagaccg ataatctgct gtttcagttc cggagacagc ataccgctca tcgccgcttt
