# Supplementary figures and images for: Divergently Transcribed ncRNAs in Escherichia coli: Refinement of the Transcription Starts Assumes Functional Diversification
Source: Front Mol Biosci. 2021 Mar 3;8:610453. doi: 10.3389/fmolb.2021.610453 (PMC7967276; doi:10.3389/fmolb.2021.610453)

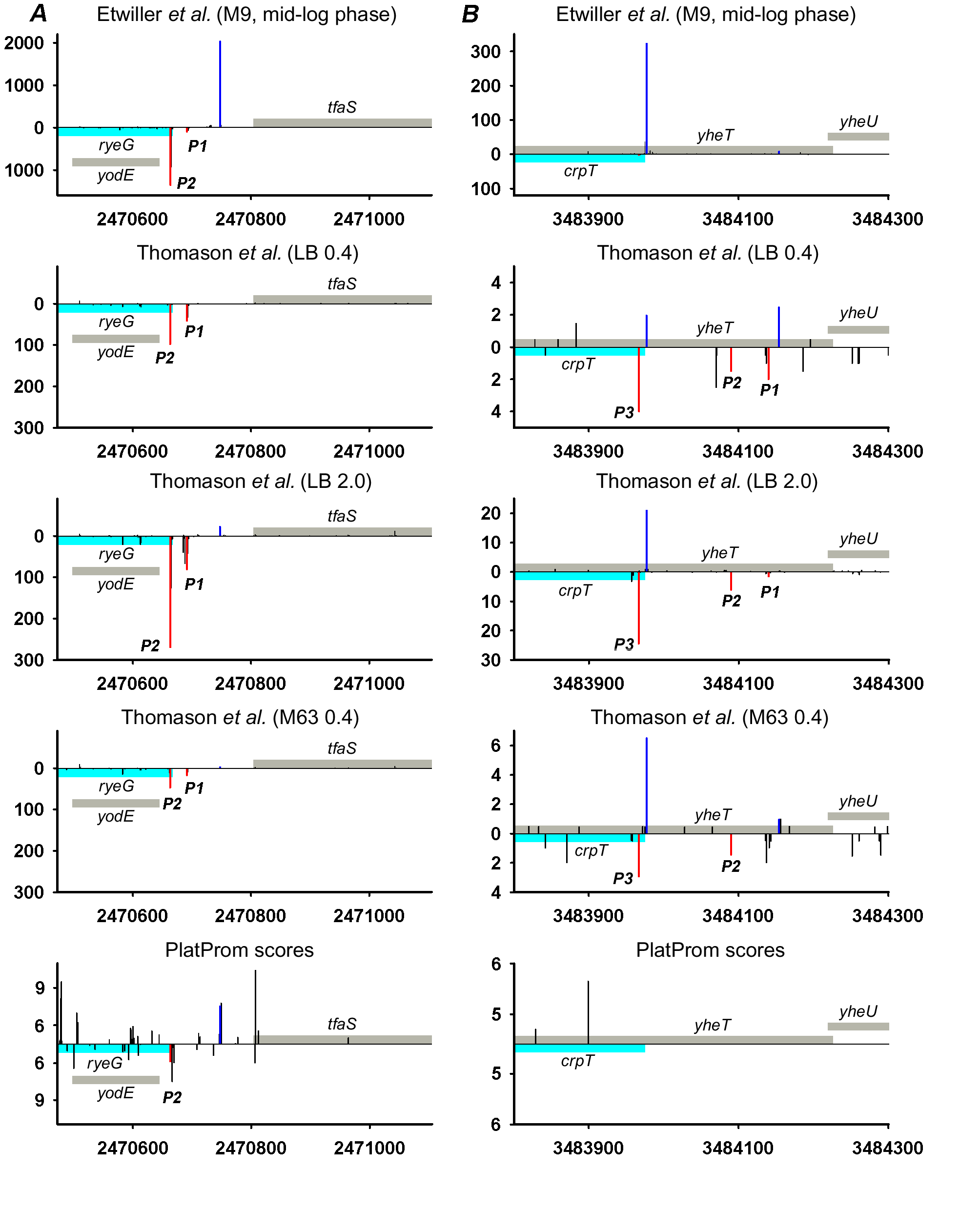

Supplement: Supplementary file 3 [file image1.tif]

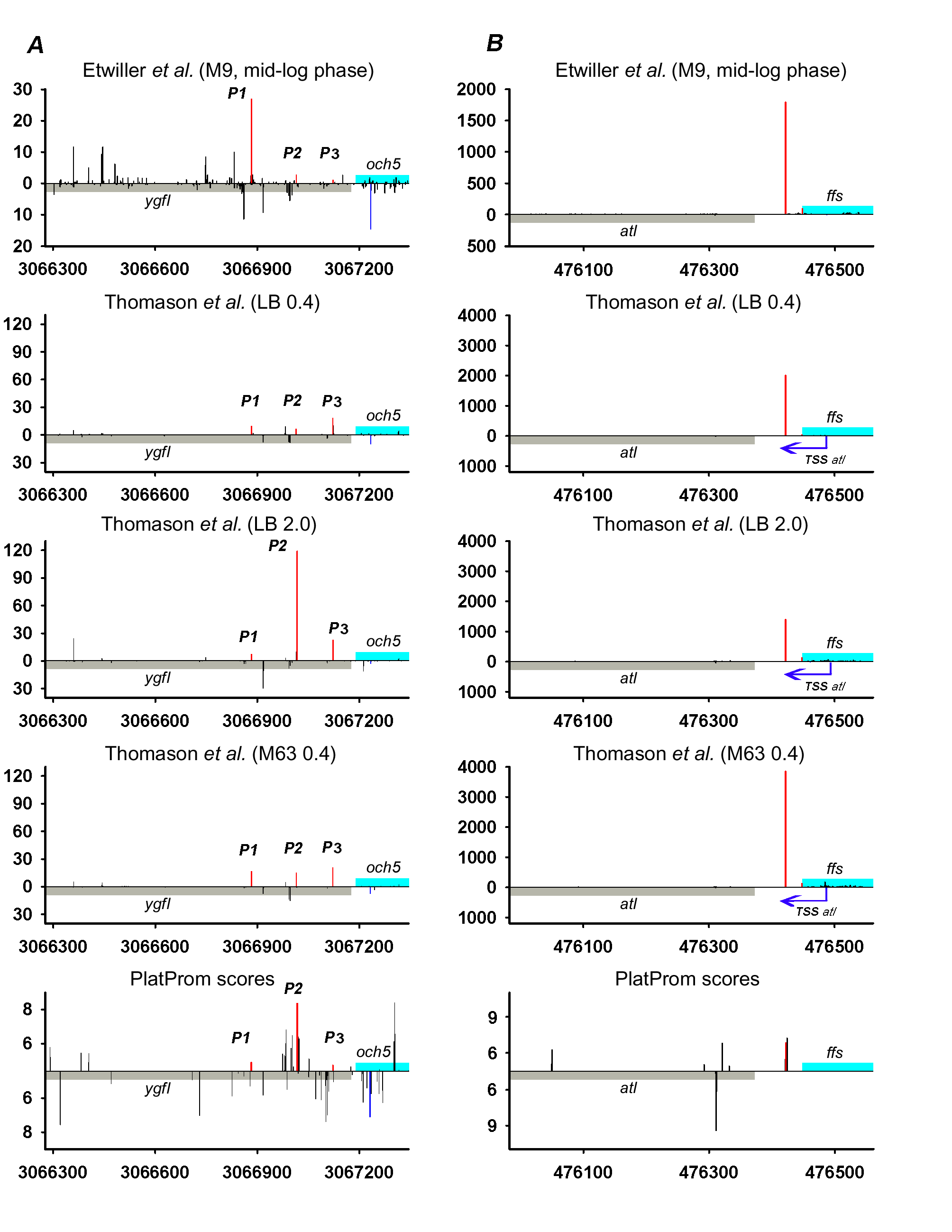

Supplement: Supplementary file 4 [file image2.tif]

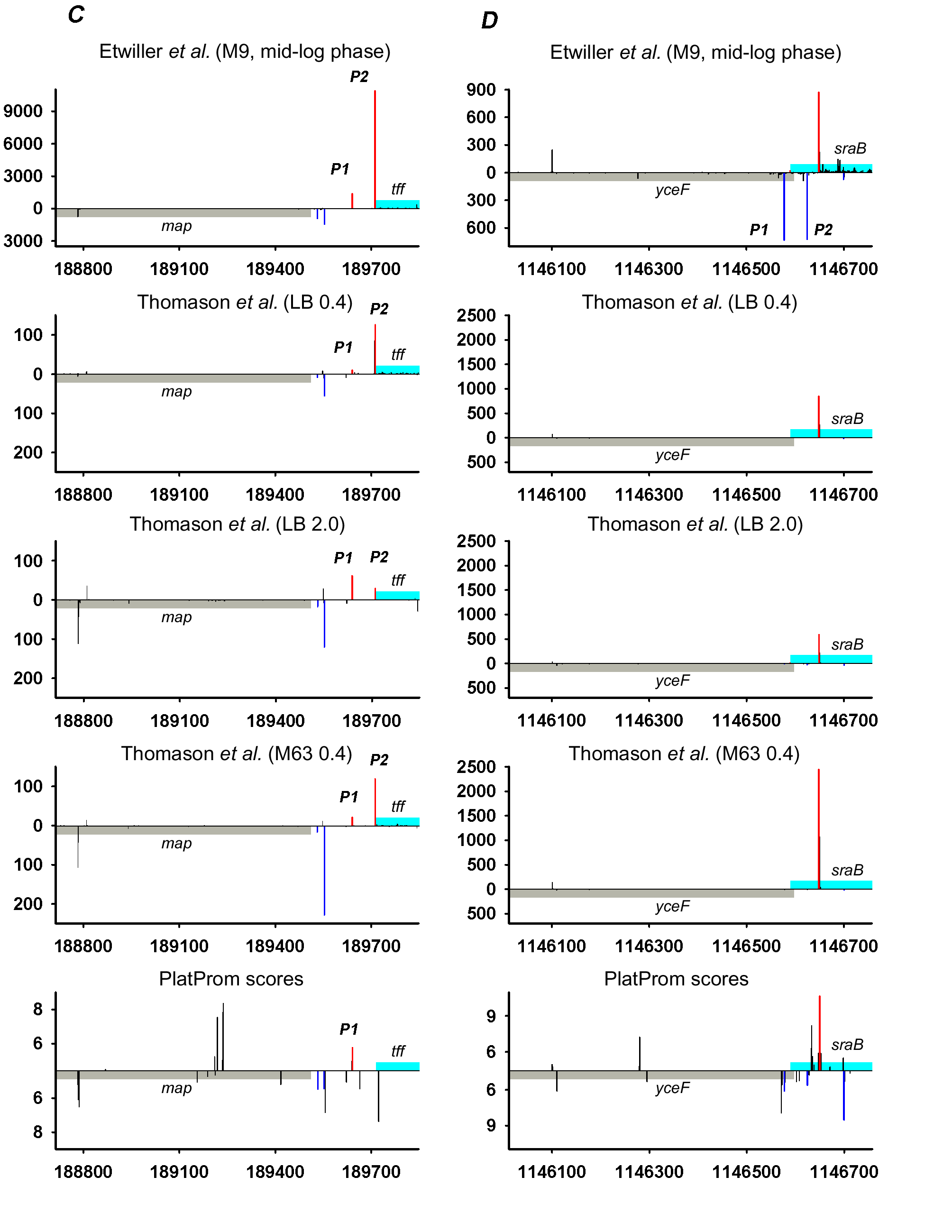

Supplement: Supplementary file 5 [file image3.tif]

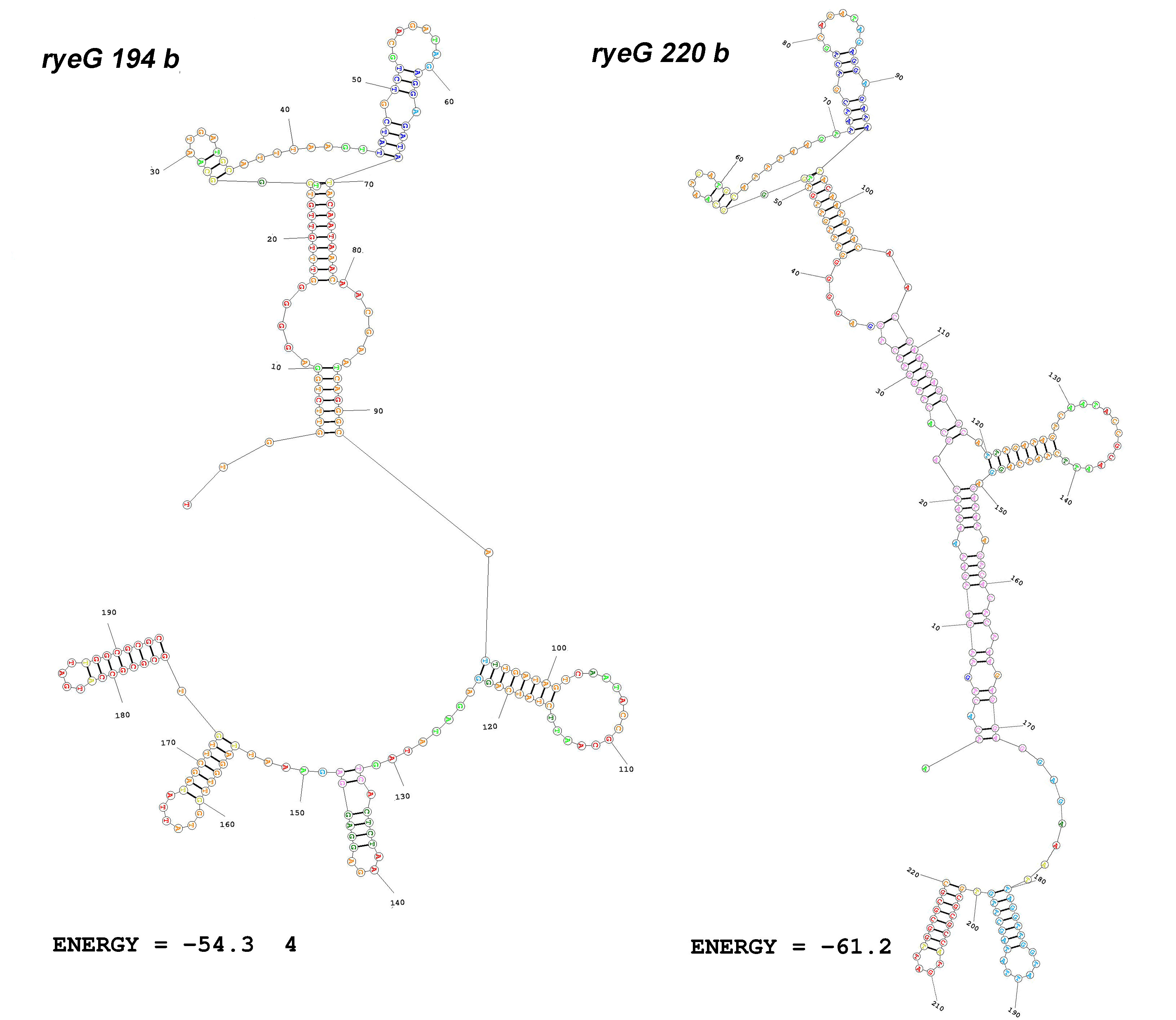

Supplement: Supplementary file 6 [file image4.tif]

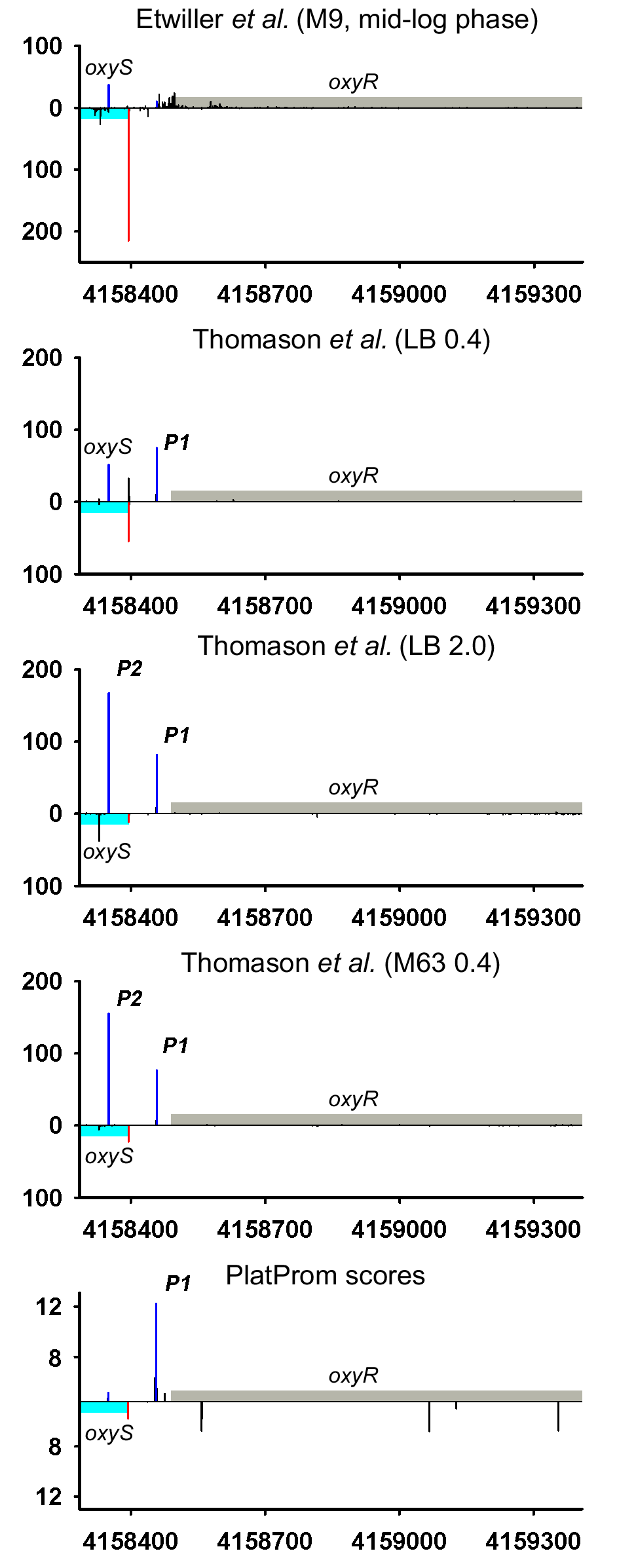

Supplement: Supplementary file 7 [file image5.tif]

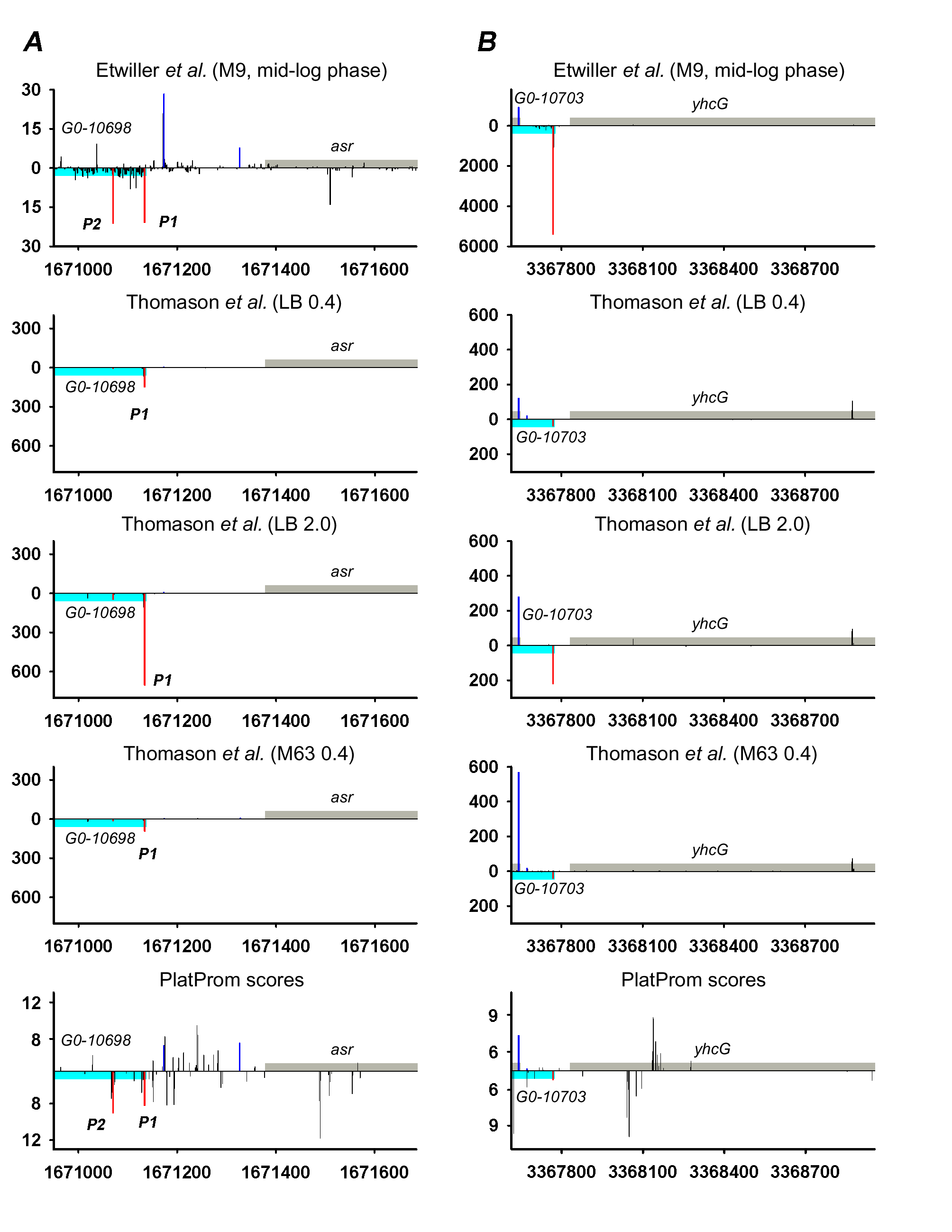

Supplement: Supplementary file 8 [file image6.tif]
